# Differences in Circulating Extracellular Vesicle and Soluble Cytokines in Older Versus Younger Breast Cancer Patients With Distinct Symptom Profiles

**DOI:** 10.3389/fgene.2022.869044

**Published:** 2022-04-25

**Authors:** Dilorom Sass, Wendy Fitzgerald, Brian S. Wolff, Isaias Torres, Glorivee Pagan-Mercado, Terri S. Armstrong, Christine Miaskowski, Leonid Margolis, Leorey Saligan, Kord M. Kober

**Affiliations:** ^1^ National Institute of Nursing Research, National Institutes of Health (NIH), Bethesda, MD, United States; ^2^ Section on Intercellular Interactions, Eunice Kennedy Shriver National Institute of Child Health and Human Development, NIH, Bethesda, MD, United States; ^3^ Neuro-Oncology Branch, Center for Cancer Research, National Cancer Institute, National Institutes of Health, Bethesda, MD, United States; ^4^ School of Nursing, University of California, San Francisco, San Francisco, CA, United States

**Keywords:** extracellular vesicles, cytokines, breast cancer, symptoms, latent class

## Abstract

Because extracellular vesicle (EV)-associated cytokines, both encapsulated and surface bound, have been associated with symptom severity, and may vary over the lifespan, they may be potential biomarkers to uncover underlying mechanisms of various conditions. This study evaluated the associations of soluble and EV-associated cytokine concentrations with distinct symptom profiles reported by 290 women with breast cancer prior to surgery. Patients were classified into older (≥60 years, *n* = 93) and younger (< 60 years, *n* = 197) cohorts within two previously identified distinct symptom severity profiles, that included pain, depressive symptoms, sleep disturbance, and fatigue (i.e., High Fatigue Low Pain and All Low). EVs were extracted using ExoQuick. Cytokine concentrations were determined using Luminex multiplex assay. Mann Whitney *U* test evaluated the differences in EV and soluble cytokine levels between symptom classes and between and within the older and younger cohorts adjusting for Karnofsky Performance Status (KPS) score, body mass index (BMI), and stage of disease. Partial correlation analyses were run between symptom severity scores and cytokine concentrations. Results of this study suggest that levels of cytokine concentrations differ between EV and soluble fractions. Several EV and soluble pro-inflammatory cytokines had positive associations with depressive symptoms and fatigue within both age cohorts and symptom profiles. In addition, in the older cohort with High Fatigue Low Pain symptom profile, EV GM-CSF concentrations were higher compared to the All Low symptom profile (*p* < 0.05). Albeit limited by a small sample size, these exploratory analyses provide new information on the association between cytokines and symptom profiles of older and younger cohorts. Of note, unique EV-associated cytokines were found in older patients and in specific symptom classes. These results suggest that EVs may be potential biomarker discovery tools. Understanding the mechanisms that underlie distinct symptom class profiles categorized by age may inform intervention trials and offer precision medicine approaches.

## Introduction

Oncology patients commonly experience co-occurring symptoms including pain, fatigue, sleep disturbance, and depression. These symptoms form clusters and are associated with decrements in quality of life (Kim et al., 2005; Miaskowski et al., 2006). Symptom clusters are thought to share common underlying mechanisms such as immune dysregulation and activation of the sympathetic nervous system (Miaskowski et al., 2017). In our previous report (Doong et al., 2015), using the three identified latent classes (i.e., All Low, High Fatigue/Low Pain, and All High), we found that younger age, poor functional status and single nucleotide polymorphisms in interleukin (IL)-6, IL-13, and tumor necrosis factor-α were associated with membership in the All High class. Other studies found associations between pro-inflammatory cytokines and a higher symptom burden in men with prostate cancer (Sass et al., 2021) and women with breast cancer (Bower et al., 2011), which suggests that discrepancies in cytokine production may be a possible mechanism that underlies the co-occurrence of common symptoms in oncology patients.

Increasing evidence suggests that health outcomes including symptom burden are worse in oncology patients with health disparities (National Cancer Institute, 2020). Age is one of the factors that can lead to worse health outcomes in women with breast cancer (Chen et al., 2016; Bagegni and Peterson, 2020). In studies of patients with heterogeneous types of cancer, findings regarding age are disparate, with several reports (Cataldo et al., 2013; Ritchie et al., 2014; Miaskowski et al., 2015) demonstrating that younger age was associated with higher symptom severity and lower quality of life. However, others (Agasi-Idenburg et al., 2017) found no age-related differences in the number and types of symptom clusters identified. In contrast, in a study that explored markers of inflammation (Alibhai S. M. H. et al., 2020), larger associations were found between fatigue severity and plasma levels of 31 cytokines in older patients with leukemia.

Increased age is associated with low grade inflammation and mild pro-inflammatory states are correlated to disease progression in the older population (Franceschi and Campisi, 2014). In addition, inflammation has been proposed as one of the potential mechanisms for increased symptom severity (Miaskowski et al., 2017). However, given the relationship between younger age and greater symptom burden (Cataldo et al., 2013; Ritchie et al., 2014; Miaskowski et al., 2015), a closer examination of age-related (younger and older) differences and cytokine variations associated with a pre-specified symptom cluster (i.e., pain, fatigue, sleep disturbance, and depression) is warranted at the present time.

An evaluation of cytokines as biomarkers for symptoms using standard cytokine assays may miss extracellular vesicle (EV)-associated cytokines due to their incorporation into the lipid bilayer or encapsulation inside EVs (Fitzgerald et al., 2018a; Margolis and Sadovsky, 2019). Thus, a comprehensive assessment of both EV-associated (surface bound and encapsulated) and soluble cytokines may provide additional information into symptom biology. EVs, which are small lipid bilayer particles that range in size from 30 nm (nm) to 1,000 nm and house rich encapsulated contents of nucleic acids and protein material, vary in composition and characteristics over the lifespan, and are increasingly being recognized as potential biomarkers of disease and its progression (Cicero et al., 2015; Robbins, 2017; Lananna and Imai, 2021).

Findings from a limited number of studies in cancer and traumatic brain injury suggest that changes in exosomal or EV-associated cytokines and proteins contribute to variations in symptoms and symptom clusters (Gill et al., 2018; Guedes et al., 2021; Sass et al., 2021). For example, in a study of men with prostate cancer who underwent radiation therapy (Sass et al., 2021), both EV and soluble forms of a variety of pro-inflammatory cytokines demonstrated positive and negative associations with psychological symptom clusters. In traumatic brain injury, EV neurofilament light chain (Guedes et al., 2021) and EV IL-10 were associated with post-traumatic stress disorder symptom severity (Gill et al., 2018).

To our knowledge, no studies have evaluated age-related differences in EV-associated cytokines (also referred as EV cytokines) in the context of a pre-specified symptom cluster. Therefore, the purposes of this study, in a sample of women who were assessed prior to breast cancer surgery, were to: investigate age-related differences in EV and soluble cytokines associated with a pre-specified symptom cluster (i.e., pain, fatigue, sleep disturbance, and depression) and explore associations between the levels of cytokines and pain, fatigue, sleep disturbance, and depressive symptom severity scores. This type of exploration may provide new insights into the utility of EVs to evaluate age-related differences in patients’ symptom experiences and associated changes in cytokine regulation.

## Materials and Methods

### Patients and Settings

This analysis is part of a larger study whose details are published elsewhere (Miaskowski et al., 2013). In brief, 398 patients were recruited from Breast Care Centers located in a Comprehensive Cancer Center, two public hospitals, and four community practices prior to their breast cancer surgery. Patients were eligible to participate if they were an adult woman (≥18 years) who would undergo breast cancer surgery on one breast; were able to read, write, and understand English; and provided written informed consent. Patients were excluded if they were having breast cancer surgery on both breasts and/or had distant metastasis at the time of diagnosis. A total of 516 patients were approached to participate, 410 were enrolled in the study (response rate 79.5%), and 398 completed the study questionnaires. Patients (*n* = 290) with complete symptom and biomarker data were included in this analysis.

### Study Procedures

Study was approved by the Committee on Human Research at the University of California, San Francisco and by the Institutional Review Boards at each of the study sites. During the patient’s preoperative visit, a clinician explained the study to the patient, determined her willingness to participate, and introduced the patient to the research nurse. The research nurse determined eligibility and obtained written informed consent prior to surgery. After the consent process, patients completed the enrollment questionnaires and blood specimens were obtained.

### Instruments

A demographic questionnaire obtained information on age, education, ethnicity, marital status, employment status, living situation, and financial status. The Karnofsky Performance Status (KPS) scale was used to evaluate patient’s functional status (Karnofsky, 1977).

Pain intensity was assessed using a 0 (no pain) to 10 (worst imaginable pain) numeric rating scale (NRS). The NRS is a valid and reliable measure to assess pain in oncology patients (Jensen, 2003).

Fatigue was assessed using the 18-item Lee Fatigue Scale (LFS) that evaluates physical fatigue and energy (Lee et al., 1991). Total fatigue scores were calculated as the mean of the 13 fatigue items. A cut-off score of ≥4.4 indicates a high level of fatigue. Cronbach’s alpha for fatigue scale was 0.96.

Sleep disturbance was evaluated using the 21-item General Sleep Disturbance Scale (GSDS) (Lee, 1992). The GSDS total score ranges from 0 to 147 with a score of ≥43.0 indicating a clinically meaningful level of sleep disturbance. Cronbach’s alpha for the GSDS total score was 0.86.

Depressive symptoms were assessed using the 20-item Center for Epidemiological Studies-Depression (CES-D) Scale. CES-D scores can range from 0 to 60, with scores of ≥16 indicating the need for a clinical evaluation (Hann et al., 1999). Cronbach’s alpha for the CES-D was 0.90.

### Latent Class Analyses

As reported previously, latent class analysis (LCA) (Vermunt and Magidson, 2002) was used to identify subgroups of patients with distinct symptom profiles (i.e., latent classes) for the pre-specified symptom cluster of pain, fatigue, sleep disturbance, and depression (Doong et al., 2015). Detailed description of phenotypic data and latent class analyses were reported previously (Doong et al., 2015) and included: All Low, High Fatigue/Low Pain, and All High classes. For this analysis, we utilized data from patients in the All Low and High Fatigue/Low Pain classes. The All High class was not included because these patients were primarily young and the sample size was small (*n* = 18). Our preliminary analysis of All Low and High Fatigue/Low Pain classes found higher levels of cytokines in All Low class with age being a significant confounder (not shown here). Therefore, the aims of this paper were to determine whether cytokine expression differed between and among the pre-specified symptom cluster classes stratified by age.

### Blood Collection and EV Isolation

Of the 398 patients enrolled, 308 provided a blood sample that was processed for plasma and stored at −80°C for additional analyses. Plasma was defrosted and centrifuged twice at 3,000 g for 15 min to produce a platelet poor plasma (PPP). EVs were isolated using ExoQuick ™ (SystemBio, Palo Alto, CA) following the manufacturer’s protocol. Starting volumes used for EV isolations varied from 230 to 500 µl per patient (average 230 µl of PPP per sample). After defrosting and two centrifugation steps, samples were incubated with 58 µl of ExoQuick for 30 min at 4°C. The ExoQuick plus PPP mixture was centrifuged at 1,500 g for 30 min to separate supernatant (EV depleted fraction) and EV enriched pellet. EV pellet was resuspended back to the starting volume with sterile phosphate buffered saline (PBS). EV depleted and EV enriched fractions were stored at −80°C for an average of 30 days (range: 10–69 days) before cytokine analyses. Laboratory staff who processed EVs and multiplex assays were blind to patients’ symptom class assignment.

EV markers were characterized using flow cytometry for size and concentration via microfluidic resistive pulse sensing using the nCS1™ particle analyzer (Supplementary Material).

### Cytokine Measurements

Concentrations of cytokines were measured in EV depleted supernatant (also referred here as soluble) and EV enriched fractions using a bead-based multiplex assay (Luminex). This in-house assay was validated previously (Fitzgerald et al., 2018b; Sass et al., 2020) and included 35 cytokines: interleukin (IL)-1α, IL-1β, IL-2, IL-4, IL-6, IL-7, IL-8, IL-10, IL-12p70, IL-13, IL-15, IL-16, IL-17, IL-18, IL-21, IL-22, granulocyte-macrophage colony-stimulating factor (GM-CSF), growth-regulated alpha (GRO-α or CXCL1), interferon-γ (IFN-γ), interferon-γ-induced protein (IP-10 or CXCL10), interferon-inducible T-cell alpha chemoattractant (ITAC or CXCL11), macrophage colony-stimulating factor (M-CSF), monocyte chemoattractant protein-1 (MCP-1 or CCL2), monokine induced by IFN-γ (MIG or CXCL9), macrophage inflammatory protein-1α (MIP-1α or CCL3), MIP-1β (CCL4), MIP-3α (CCL20), transforming growth factor beta (TGF-β), tumor necrosis factor-α (TNF-α), IL-1 receptor antagonist (IL-1RA), MIP-3β, programmed death-ligand 1 (PD-L1), TNF-related apoptosis-inducing ligand (TRAIL), vascular endothelial growth factor (VEGF), and regulated on activation normally T-cell expressed and secreted (RANTES or CCL5). For the final analysis, 32 cytokines were included and three (RANTES, IL-13, IL-21) were excluded due to high background and being below or above limits of detection.

Magnetic beads were coupled with protein specific capture antibodies. Standards (unlysed or lysed with 0.1% Triton X-100), lysed EVs (to measure both surface and encapsulated EV cytokines), and EVdepleted supernatants (unlysed) were diluted in assay buffer and incubated with bead mixes overnight at 4°C. The next day, the plates were washed twice and incubated with cytokine specific biotinylated antibodies for 1 hour. After the incubation, plates were washed again and incubated with streptavidin-phycoerythrin (16 μg/ml) in PBS (Thermo Fisher, Waltham, MA). After final washes, 60 µl of PBS were added to each well to resuspend beads. Cytokine measurements were performed on a Luminex 200 System and analyzed with Bioplex Manager software (BioRad, Hercules, CA). To account for dilution by ExoQuick ™ reagent, we adjusted final concentrations of soluble analytes. Total EV-associated cytokines (surface and internal EV cytokines) were reported by measurement of lysed EVs. Lower limits of detection (LLOD) and standard curves were determined using 5-parameter logistic regression (Cumberland et al., 2015). Data on LLOD for each analyte and descriptive statistics for cytokine concentrations are included in Supplementary Table S1 and Supplementary Table S2

### Statistical Analyses

Descriptive statistics and frequency distributions were generated for the sample characteristics (*n* = 290) and symptom data. As noted previously, patients in the ALL Low and High Fatigue/Low Pain classes were evaluated. Patients in each of these two classes were categorized into younger (< 60) and older (≥60) age cohorts using the World Health Organization age cut off for elderly (World Health Organization, 2015). Descriptive statistics were calculated for the demographic and clinical characteristics using SPSS Grad Pack version 28.0 (IBM Corporation, Armonk, NY). Figures were created using GraphPad Prism version 8.4.3 (for Windows, GraphPad Software, San Diego, California United States, www.graphpad.com) and *Python* version 3.9.6 (*Python* Software Foundation, Beaverton, OR). Partial correlation analyses were conducted using pingouin version 0.4.0 (“partial_corr”). We tested the data for a Gaussian distribution using histograms, and the skewness and kurtosis statistics (Supplementary Table S2). Concentrations of EV and soluble cytokines were log base 2 transformed to reduce skewness.

Due to non-normal distribution of the variables and small sample sizes, non-parametric two-tailed Mann Whitney U tests were done to evaluate for differences between the classes stratified by age. Effect sizes for significant cytokines identified by two group analysis were conducted using $\eta^{2}=\frac{Z^{2}}{\left(N-1\right)}\;$ . A *p*-value of ≤0.05 was considered statistically significant. Demographic and clinical characteristics that were significant in the bivariate tests were included as covariates in the logistic regression models. The overall model fit was evaluated with area under the receiver operating characteristics curve (Hosmer Jr et al., 2013). Associations between fatigue, sleep disturbance, and depression scores and levels of cytokines were evaluated using partial correlation analysis with concentrations below the LLOD removed from the analyses. Pain was excluded from the bivariate correlations because of the small number of patients who had pain prior to surgery (Table 1). Due to exploratory nature of analyses, corrections for multiple comparisons were not performed.

**TABLE 1:** Demographic and clinical characteristics.

Characteristics	Older cohort (≥60 years of Age)			Younger cohort (< 60 years of Age)		
	All low (*n* = 74)	High fatigue/Low pain (*n* = 19)	*P*	All low (*n* = 113)	High fatigue/Low pain (*n* = 84)	*p*
	Median (IQR)	Median (IQR)		Median (IQR)	Median (IQR)	
Age (years)	66.9 (63.8, 73.0)	64.4 (62.4, 67.5)	0.050	49.9 (45.0, 55.0)	49.7 (44.1, 54.6)	0.393
Education (years)	16.0 (13.0, 17.0)	15.0 (12.0, 16.0)	0.132	16.0 (14.0, 18.0)	16.0 (15.0, 18.0)	0.243
KPS score	100.0 (100.0, 100.0)	90.0 (90.0, 100.0)	< 0.001**	100.0 (90.0, 100.0)	90.0 (80.0, 100.0)	0.007*
BMI (kg/m^2^)	25.6 (22.5, 29.0)	29.5 (24.2, 34.0)	0.047*	24.3 (22.1, 28.6)	24.8 (22.8, 29.4)	0.536
Self-administered comorbidity questionnaire	4.0 (2.8, 6.0)	6.0 (4.0, 7.0)	0.070	3.0 (2.0, 5.0)	3.0 (2.0, 6.0)	0.321
	n (%)	n (%)		n (%)	n (%)	
Marital status			0.563			0.233
Married/partnered	36 (48.6)	9 (47.4)		47 (41.6)	27 (32.1)	
Single	38 (51.4)	10 (52.6)		66 (58.4)	56 (66.7)	
Ethnicity			0.132			0.051
White	54 (73.0)	11 (57.9)		73 (65.2)	65 (78.3)	
Black	6 (8.1)	0 (0.0)		7 (6.3)	5 (6.0)	
Asian	6 (8.1)	4 (21.1)		20 (17.9)	4 (4.8)	
Hispanic/Mixed	8 (10.8)	4 (21.1)		12 (10.7)	9 (10.8)	
Disease stage			0.017*			0.415
Stage 0	13 (17.6)	2 (10.5)		26 (23.0)	14 (16.7)	
Stage I	39 (52.7)	6 (31.6)		35 (31.0)	35 (41.7)	
Stage IIA/IIB	20 (27.0)	7 (36.8)		43 (38.1)	30 (35.7)	
Stage IIIA/IIIB, IV	2 (2.7)	4 (21.1)		9 (8.0)	5 (6.0)	
Estrogen receptor status			0.477			0.032*
Negative	10 (13.7)	4 (21.1)		22 (19.5)	28 (33.3)	
Positive	63 (86.3)	15 (78.9)		91 (80.5)	56 (66.7)	
Progesterone receptor status			0.234			0.081
Negative	16 (21.9)	7 (36.8)		27 (23.9)	30 (35.7)	
Positive	57 (78.1%)	12 (63.2%)		86 (76.1)	54 (64.3)	
On hormone replacement therapy prior to surgery (no)	54 (73.0)	14 (73.7)	1.000	101 (90.2)	69 (83.1)	0.193
Received neoadjuvant therapy (no)	70 (94.6)	16 (84.2)	0.148	87 (77.0)	58 (69.0)	0.253
Type of surgery						0.040*
Breast conservation	63 (85.1)	16 (84.2)		93 (82.3)	58 (69.0)	
Mastectomy	11 (14.9)	3 (15.8)		20 (17.7)	26 (31.0)	
Location of tumor			0.304			0.774
Right breast	35 (47.3)	12 (63.2)		54 (47.8)	38 (45.2)	
Left breast	39 (52.7)	7 (36.8)		59 (52.2)	46 (54.8)	
Occurrence of pain (n (%))	6 (8.1)	4 (21.1)	0.608	34 (30.0)	22 (26.2)	0.421
LFS score	1.0 (0.2, 2.1)	5.6 (4.2, 6.7)	< 0.001**	1.5 (0.6, 2.6)	5.4 (4.7, 6.7)	< 0.001**
CES-D score	9.0 (3.2, 13.0)	17.0 (12.0, 22.2)	< 0.001**	10.0 (4.2, 17.0)	14.3 (9.3, 21.0)	< 0.001**
GSDS score	32.5 (23.1, 46.2)	64.0 (43.0, 76.7)	< 0.001**	40.9 (27.0, 55.6)	59.8 (44.1, 76.65)	< 0.001**

Data presented median (IQR, range), or *n* (%) for categorical variables.

BMI = body mass index, CES-D = Center for Epidemiological Studies-Depression Scale, GSDS = General Sleep Disturbance Scale, kg = kilograms, KPS = Karnofsky Performance Status, LFS = Lee Fatigue Scale, m2 = meters squared.

## Results

### Demographic, Clinical, and Symptom Characteristics

As shown in Table 1, of the 290 women included in this analysis, 93 were in the older (≥60 years of age) and 197 in the younger (< 60 years of age) cohorts. Among the older cohort, 19 were in the High Fatigue/Low Pain class and 74 were in the All Low class. Among the younger cohort, 84 were in the High Fatigue/Low Pain class and 113 were in the All Low class.

In the older cohort, the High Fatigue/Low Pain class had a lower KPS score, a higher body mass index (BMI), more advanced disease, and higher fatigue, depression, and sleep disturbance scores compared to the All Low class. In the younger cohort, the High Fatigue/Low Pain class had a lower KPS score, was more likely to be estrogen receptor positive, less likely to have breast conservation surgery, and had higher fatigue, depression, and sleep disturbance scores compared to the All Low class.

### Flow Cytometry Verification of EVs

Flow cytometry analysis of four representative patients’ EVs demonstrated that EVs contained lipid components and EV-specific protein components. EVs demonstrated 69.1 ± 1.9% positivity for CD9, 73.9 ± 2.1% positivity for CD63, and 72.3 ± 2.3% positivity for CD81. EV-depleted fractions demonstrated similar amounts of these markers, but the amount of EVs was only 8.4 ± 1.5% than what was observed in EV-enriched fractions (Supplementary Figure S1). A lack of non-EV markers was verified in the EV fractions: 3.6 ± 0.4% positivity for apolipoprotein A and 2.4 ± 0.1% positivity for apolipoprotein B, whereas EV-depleted fractions were 7.3 ± 0.5% and 19.8 ± 1.8% positive, respectively (Supplementary Figure S2). CD31, a platelet marker, was observed on EVs (49.6 ± 4.0% positivity) in the EV-enriched fraction, and on 15.3 ± 3.3% of events in the EV-depleted fraction but these events were much less abundant (15.5 ± 3.8%) than that in the EV-enriched fraction) (Supplementary Figure S3).

### Size and Concentration of EVs

A total of 30 EV samples (about 10% of the total N used in this analysis) were processed in the nCS1™ particle analyzer. Four of the samples were excluded because of significant clogging during processing. The average concentration of vesicles in the 26 samples was 8.47 E+12 (SD = 9.7 E+12) per ml. The average size of particles was 83.8 (SD = 4.3) nm (Supplementary Figure S4).

### Percent Distributions of Cytokines Within EVs in the Total Sample

**FIGURE 1:**
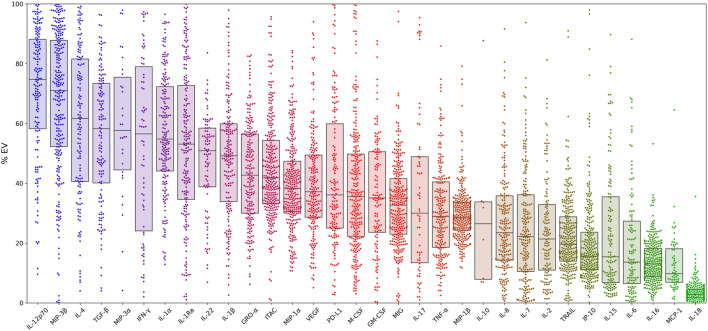
Percent distributions of 32 cytokines within extracellular vesicles for the entire cohort. Cytokine concentrations for all patients are calculated as the EV concentration divided by the sum of the EV and soluble concentrations. Box plot overlays show the medians and interquartile ranges. Samples with concentrations at the lower limit of detection (LLOD) were excluded from the analysis. In addition, three cytokines (RANTES, IL-13, IL-21) were excluded from analysis due to concentrations being above or below detection limits and/or due to high background. Abbreviations: **α** = alpha, β = beta , EV = extracellular vesicle, GM-CSF = granulocyte-macrophage colony-stimulating factor, GRO-α = growth-regulated alpha, IFN-γ = interferon-γ, IP-10 = interferon-γ-induced protein, IL = interleukin, ITAC = interferon-inducible T-cell alpha chemoattractant, M-CSF = macrophage colony-stimulating factor, MCP-1 = monocyte chemoattractant protein-1, MIG = monokine induced by IFN-γ, MIP-1α = macrophage inflammatory protein-1α, PD-L1= programmed death-ligand 1, RA = receptor antagonist, TGF-β = transforming growth factor beta, TNF-α = tumor necrosis factor-α, TRAIL = TNF- related apoptosis-inducing ligand, VEGF = vascular endothelial growth factor.

Relative cytokine concentrations in the EV pellet and supernatant (soluble) for the total sample are presented in Figure 1. Cytokine concentrations were calculated as the EV cytokine concentration divided by the total (sum of EV and soluble) concentration. MCP-1 and IL-18 were found primarily in the soluble fraction. Other cytokines (i.e.IL-12p70 and MIP-3β), showed the highest concentrations in the EV fraction.

### Differences in the Levels of EV and Soluble Cytokines Between High Fatigue/Low Pain and All Low Classes Stratified by Age

**FIGURE 2:**
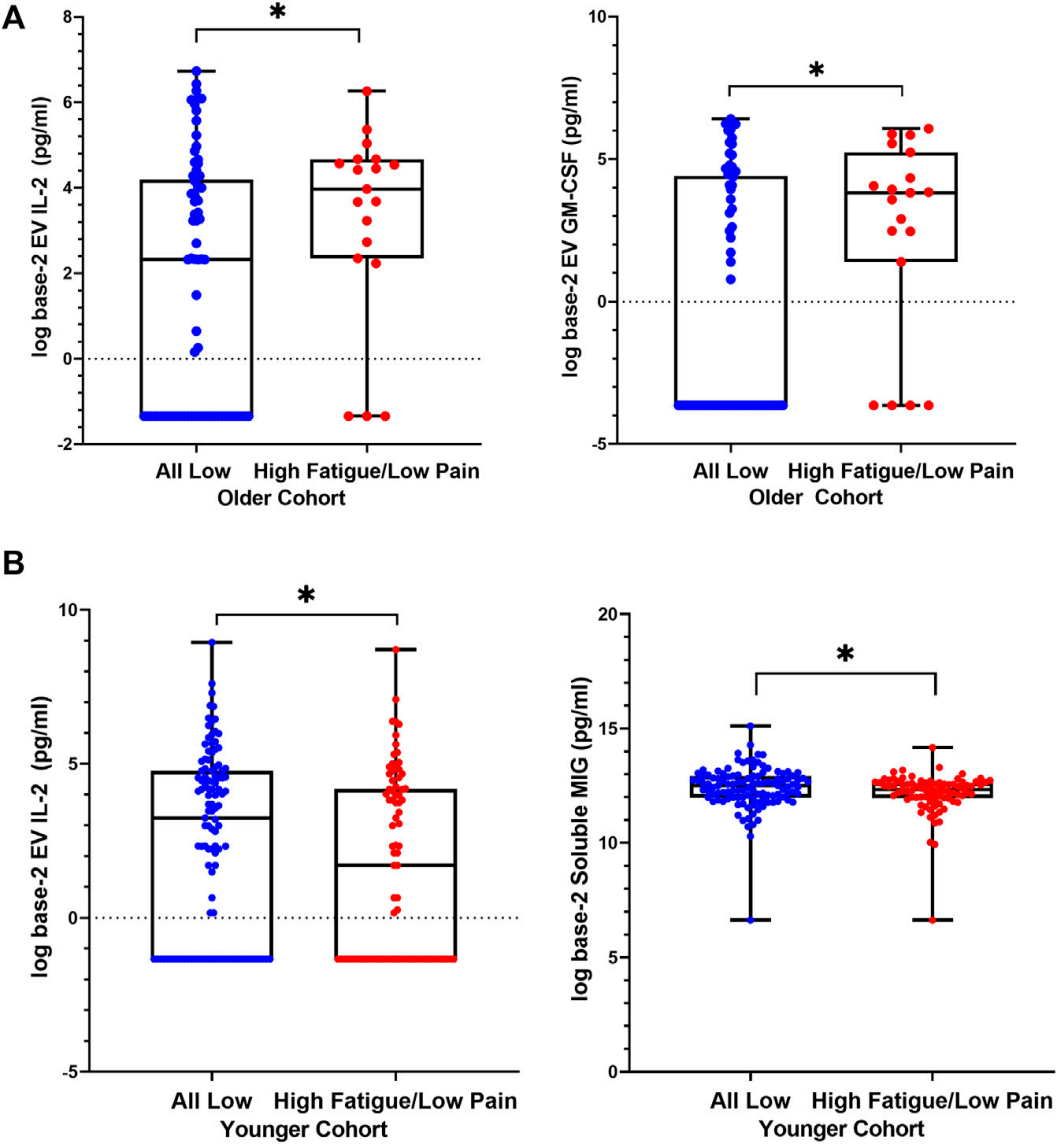
Differences in levels of extracellular vesicle-associated cytokines between High Fatigue/Low Pain versus All Low classes. Distributions are represented using boxplots, showing median, IQR, and maximum and minimum. **(A)** In older cohort, Mann-Whitney U tests comparing the two classes showed significantly higher EV IL-2 (*p* = 0.032) levels in the High Fatigue/Low Pain class (median = 3.97, IQR 2.3–4.7) compared to the All Low class (median = 2.3, IQR −1.3–4.2). Concentrations of EV GM-CSF (*p* = 0.025) were significantly elevated in the High Fatigue/Low Pain class (median = 3.8, IQR 1.4–5.2) compared to the All Low class (median = −3.6, IQR −3.6–4.4). **(B)** In younger cohort, Mann-Whitney *U* test comparing the two classes showed significantly higher EV IL-2 (*p* = 0.040) levels in the All Low class (median = 3.2, IQR −1.3–4.7) compared to the High Fatigue/Low Pain class (median = 1.6, IQR −1.3–4.2). Concentrations of soluble MIG (*p* = 0.043) were significantly elevated in the All Low class (median = 12.5, IQR 11.9–12.9) compared to the High Fatigue Low Pain class (median = 12.3, IQR 11.9–12.6). Abbreviations: EV = extracellular vesicle, GM-CSF = granulocyte-macrophage colony-stimulating factor, IL = interleukin, MIG = monokine induced by IFN-γ.

Within the older cohort, in High Fatigue/Low Pain class, higher concentrations of EV IL-2 and EV GM-CSF were observed compared to the All Low class (Table 2A; Figure 2A). After controlling for KPS score, BMI, and stage of disease, EV IL-2 concentration was marginally significant (*p* = 0.053, η^2^ = 0.050) and GM-CSF remained significant (*p* = 0.019, η^2^ = 0.055). The models were assessed to have excellent discrimination (0.80 < *AUC* < 0.90) (Table 2).

**TABLE 2:** Logistic regression models controlling for significant covariates in older **(A)** and younger **(B)** cohorts.

(A) logistic Regression Model Controlling for Significant Covariates in Older Cohort				
Predictors	Coefficient (B)	*SE*	*OR*	*p*
EV IL-2	0.37	0.134	1.295	0.053
KPS	−0.11	0.043	0.892	0.008
BMI	0.09	0.044	1.097	0.034
Stage of disease*				0.172
Stage 0	−2.29	1.266	0.100	0.069
Stage I	−2.42	1.106	0.089	0.029
Stages IIA and IIB	−1.91	1.083	0.148	0.077
Overall model fit: AUC of the ROC = 0.828				
EV GM-CSF	0.21	0.089	1.233	0.019*
KPS	−0.10	0.048	0.905	0.036
BMI	0.11	0.046	1.117	0.016
Stage of disease*				0.101
Stage 0	−2.94	1.398	0.053	0.035
Stage I	−2.95	1.209	0.052	0.015
Stages IIA and IIB	−2.31	1.176	0.099	0.049
Overall model fit: AUC of the ROC = 0.852				
**(B) Logistic regression model controlling for significant covariates in younger cohort**				
EV IL-2	−0.09	0.051	0.914	0.081
KPS	−0.03	0.015	0.968	0.029
Estrogen receptor status	−0.57	0.348	1.760	0.104
Overall model fit: AUC of the ROC = 0.653				
Soluble MIG	−0.36	0.188	0.695	0.053
KPS	−0.04	0.015	0.962	0.009
Estrogen receptor status	−0.44	0.353	1.553	0.212
Overall model fit: AUC of the ROC = 0.657				

Abbreviations: AUC = area under the curve, BMI = body mass index, EV = extracellular vesicle, GM-CSF = granulocyte-macrophage colony-stimulating factor, IL = interleukin, KPS = Karnofsky Performance Status, MIG = monokine induced by IFN-γ, OR = odds ratio, ROC = receiver operating characteristics, SE = standard error.

Note: “type of surgery” was not controlled in the younger cohort model, because blood is collected prior to surgery.

**(A)** Logistic regression model controlling for KPS, BMI, stage of disease (Stage 0, Stage I, Stages IIA, IIB, and Stages IIIA, IIIB, IIIC, and IV), log base 2 transformed EV IL-2, and GM-CSF, in the older cohort. EV IL-2, remains marginally significant (overall model fit: AUC, of the ROC, 0.825), and EV GM-CSF, remains significant (overall model fit: AUC, of the ROC, 0.840) **(B)** Logistic regression model controlling for KPS, and estrogen receptor status (negative, positive), log base 2 transformed EV IL-2, and soluble MIG, in younger cohort. Soluble MIG, remains marginally significant (overall model fit: AUC, of the ROC, 0.657), and EV, Il-2 was no longer significant (overall model fit: AUC, of the ROC, 0.653).

In the younger cohort, an opposite trend was observed. In the High Fatigue/Low Pain class, EV IL-2 and soluble MIG concentrations were significantly lower compared to All Low class (Table 2B and Figure 2B). After controlling for KPS score and estrogen receptor status, EV IL-2 was no longer significant (*p* = 0.081, η^2^ = 0.021) and soluble MIG was marginally significant (*p* = 0.053, η^2^ = 0.021). The models were assessed to have nearing acceptable discrimination (0.50 < *AUC* < 0.70) (Table 2).

### Partial Correlation Results Between Cytokine Concentrations and Depression, Fatigue, and Sleep Disturbances

**FIGURE 3:**
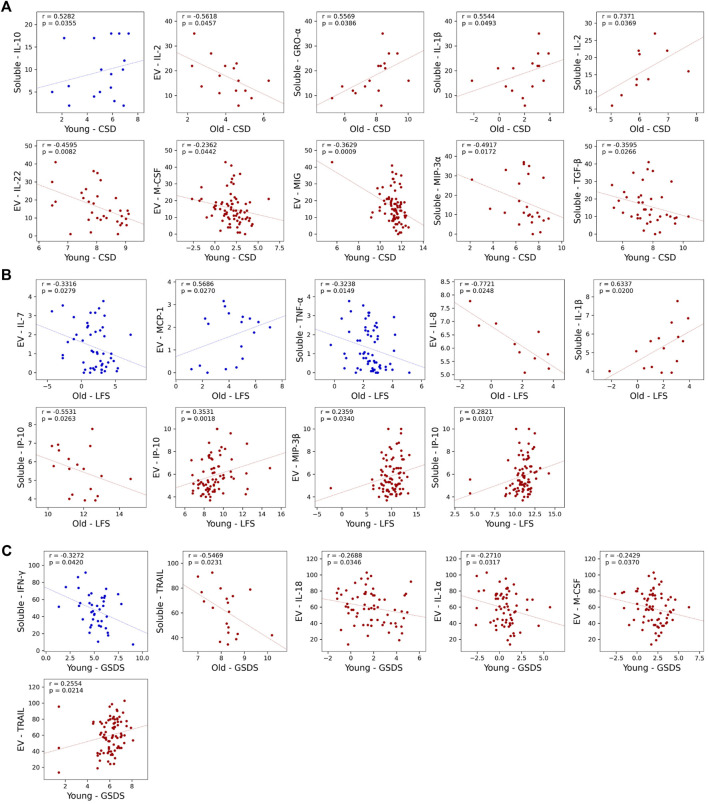
Scatterplots showing statistically significant partial correlations between measured cytokine concentrations and three of the symptoms at enrollment: Center for Epidemiologic Studies-Depression Scale (CES-D) **(A)**, Lee Fatigue Scale (LFS) **(B)**, and General Sleep Disturbance Scale (GSDS) **(C)**. Partial correlations are calculated using Spearman correlation, with Karnofsky Performance Status (KPS) scale used as a covariate in the younger cohort and both KPS and body mass index (BMI) used as a covariate in the older cohort. Cytokine concentrations outside of the detection range were excluded from analysis, as were groups with *n* < 10. Data from High Fatigue/Low Pain classes are shown in red and data from All Low classes are shown in blue. Abbreviations: α = alpha, β = beta , CES-D = Center for Epidemiological Studies-Depression Scale, EV = extracellular vesicle, GSDS = General Sleep Disturbance Scale, GRO-α = growth-regulated alpha, IFN-γ = interferon-γ, IL= interleukin, IP-10 = interferon-γ-induced protein, LFS= Lee Fatigue Scale, MCP-1 = monocyte chemoattractant protein-1, MIP = macrophage inflammatory protein, M-CSF = macrophage colony-stimulating factor, MIG = monokine induced by IFN-γ, TGF-β = transforming growth factor beta, TNF-α = tumor necrosis factor-α, TRAIL = TNF- related apoptosis-inducing ligand.

Associations with individual symptoms were performed next (Figure 3 **A-C,** Table 3, Supplementary Tables S3–S4). In the older cohort within the High Fatigue/Low Pain class, after adjusting for BMI and KPS scores, depression scores were positively correlated with soluble GRO-α (*r* = 0.56, *p* = 0.039, n = 16), IL-1β (*r* = 0.55, *p* = 0.049, *n* = 15), and IL-2 (*r* = 0.74, *p* = 0.037, *n* = 10) and negatively correlated with EV IL-2 (*r* = −0.56, *p* = 0.046, *n* = 15). Fatigue scores were positively correlated with soluble IL-1β (*r* = 0.63, *p* = 0.020, *n* = 15) and negatively correlated with EV IL-8 (*r* = =−0.77, *p* = 0.025, *n* = 10) and soluble IP10 (*r* = −0.55, *p* = 0.026, *n* = 18). Sleep disturbances scores were negatively correlated with soluble TRAIL (*r* = −0.55, *p* = 0.023, *n* = 19).

**TABLE 3:** Significant correlations between cytokine concentrations and severity of depression, fatigue, and sleep disturbance stratified by age cohorts and symptom classes.

		Older cohort						Younger cohort					
MeasuresDepression		High fatigue/Low pain			All low			High/Fatigue low pain			All low		
**Cytokine**		**r**	**P**	**n**	**r**	p	n	**r**	**p**	**n**	**r**	**p**	**n**
IL-22	EV	NR	NR	NR	0.22	0.314	25	−0.46	0.008*	33	−0.13	0.336	48
M-CSF	EV	0.23	0.383	18	0.04	0.749	56	−0.24	0.044*	74	−0.07	0.485	101
MIG	EV	−0.26	0.331	18	−0.10	0.435	67	−0.36	0.001*	81	−0.09	0.365	109
IL-2	EV	−0.56	0.046*	15	0.10	0.554	39	−0.07	0.652	46	0.19	0.090	75
IL-1β	Sol	0.55	0.049*	15	0.03	0.829	45	−0.14	0.284	61	0.21	0.061	81
Gro-α	Sol	0.56	0.039*	16	0.02	0.909	55	−0.13	0.315	61	−0.05	0.670	87
IL-10	Sol	NR	NR	NR	−0.25	0.480	12	NR	NR	NR	0.53	0.035*	17
IL-2	Sol	0.74	0.037*	10	0.03	0.845	36	−0.14	0.328	49	0.07	0.559	65
MIP-3α	Sol	NR	NR	NR	0.01	0.960	17	−0.49	0.017*	24	−0.02	0.907	31
TGF-β	Sol	NR	NR	NR	0.11	0.542	36	−0.36	0.027*	39	0.12	0.360	57
**Fatigue**													
IL-7	EV	−0.30	0.335	14	−0.33	0.028*	46	0.07	0.636	55	−0.01	0.947	79
IL-8	EV	−0.77	0.025*	10	−0.12	0.439	47	0.19	0.184	54	−0.02	0.893	78
IP-10	EV	−0.33	0.224	17	0.02	0.878	64	0.35	0.002*	77	0.08	0.424	103
MCP-1	EV	NR	NR	NR	0.57	0.026*	17	-0.09	0.734	15	−0.25	0.281	21
MIP-3β	EV	−0.20	0.452	18	0.00	0.996	65	0.24	0.034*	82	0.01	0.932	107
IL-1β	Sol	0.63	0.020*	15	−0.05	0.745	45	0.08	0.536	62	−0.06	0.616	81
IP-10	Sol	−0.55	0.026*	18	0.01	0.968	65	0.28	0.011*	82	0.09	0.372	107
TNF-α	Sol	−0.11	0.714	16	−0.32	0.015*	58	0.10	0.417	64	−0.02	0.825	88
**Sleep Disturbances**													
IL-18	EV	−0.03	0.913	16	0.05	0.699	54	−0.27	0.035*	63	0.08	0.442	89
IL-1α	EV	−0.07	0.836	14	−0.02	0.909	53	−0.27	0.032*	64	−0.18	0.112	80
IL-22	EV	NR	NR	NR	0.08	0.705	25	−0.30	0.089	34	−0.04	0.791	48
M-CSF	EV	−0.03	0.921	19	−0.08	0.571	55	−0.24	0.037*	75	−0.02	0.858	100
TRAIL	EV	−0.37	0.143	19	−0.08	0.522	66	0.26	0.021*	82	−0.05	0.616	107
IFN-γ	Sol	NR	NR	NR	−0.37	0.059	28	0.19	0.306	30	−0.33	0.042*	40
TRAIL	Sol	−0.55	0.023*	19	−0.07	0.558	66	0.20	0.071	82	0.04	0.697	108

**Note**: Partial correlation analysis with concentrations below the lower limit of detection (LLOD) and groups with *n* < 10 removed from the analyses as not reported (NR) and controlling for significant covariates. Comprehensive results are presented in Supplementary tables 3 and 4.

**Abbreviations:** α = alpha, β = beta , EV = extracellular vesicle, IL = interleukin, GRO-α = growth-regulated alpha, IFN-γ = interferon-γ (gamma, IP-10 = interferon-γ-induced protein, M-CSF = macrophage colony-stimulating factor, MCP-1 = monocyte chemoattractant protein-1, MIG = monokine induced by IFN-γ, MIP = macrophage inflammatory protein, NR = not reported, Sol = soluble, TGF-β = transforming growth factor beta, TNF-α = tumor necrosis factor-α, TRAIL = TNF- related apoptosis-inducing ligand.

In contrast, within the All Low class, no significant associations were found for depression or sleep disturbances scores. However, fatigue scores were positively correlated with EV MCP-1 (*r* = 0.57, *p* = 0.026, *n* = 17) and negatively correlated with EV IL-7 (*r* = −0.33, *p* = 0.028, *n* = 46) and soluble TNF-α (*r* = −0.32, *p* = 0.015, *n* = 58).

In the younger cohort within High Fatigue/Low Pain class, after adjusting for KPS scores, depression scores were negatively correlated with EV IL-22 (*r* = −0.46, *p* = 0.008, *n* = 33), EV M-CSF (*r* = −0.24, *p* = 0.044, *n* = 74), EV MIG (*r* = −0.36, *p* = 0.001, *n* = 81), soluble MIP-3α (*r* = −0.49, *p* = 0.017, *n* = 24), and soluble TGF-β (*r* = −0.36, *p* = 0.027, *n* = 39). Fatigue scores were positively correlated with EV IP-10 (*r* = 0.35, *p* = 0.002, *n* = 77), EV MIP-3β (*r* = 0.24, *p* = 0.034, *n* = 82), and soluble IP-10 (*r* = 0.28, *p* = 0.011, *n* = 82). Sleep disturbances scores were positively correlated with EV TRAIL (*r* = 0.26, *p* = 0.021, *n* = 82) and negatively correlated with EV IL-18 (*r* = −0.27, *p* = 0.035, *n* = 63), IL-1α (*r* = −0.27, *p* = 0.032, *n* = 64), and M-CSF (*r* = −0.24, *p* = 0.037, *n* = 75).

In contrast, within the All Low class, depression scores were positively correlated with soluble IL-10 (*r* = 0.53, *p* = 0.035, *n* = 17) and sleep disturbances scores negatively correlated with soluble IFN-γ (*r* = −0.33, *p* = 0.042, *n* = 40). No significant associations were found with fatigue scores.

## Discussion

To our knowledge, this study is the first age-based comparison of associations between EV and soluble cytokines in patients with breast cancer who were grouped into two distinct pre-specified symptom cluster classes. In the older cohort, concentrations of EV GM-CSF (significantly) and EV IL-2 (marginally) were higher in the higher symptom burden class (i.e., High Fatigue/Low Pain). In contrast, in the younger cohort, lower EV IL-2 and soluble MIG concentrations were found to be marginally significant in the higher symptom burden class. In the bivariate correlation analyses for the older cohort in the higher symptom burden class, higher depressive symptom scores were associated with higher levels of GRO-α, IL-1β, and IL-2. However, for the younger cohort with a higher symptom burden, higher depressive symptoms were associated with lower levels of IL-22, M-CSF, MIG, MIP-3α, and TGF-β. Of note, higher levels of fatigue were associated with higher levels of proinflammatory cytokines in both older (IL-1β) and younger (IP-10 and MIP-3β) women with a higher symptom burden. We hypothesize that the differences in the cytokine relationships between the age cohorts with different levels of symptom burden may be related to variation in immune pathways associated with aging, cancer, and/or symptom biology.

Our primary results compared cytokine differences between two symptom classes and age cohorts. GM-CSF, produced by macrophages and T cells stimulates the growth and production of granulocytes, macrophages, and dendritic cells (Murphy and Weaver, 2016). Our finding in our older cohort that a higher symptom burden was associated with elevated levels of EV GM-CSF differed from another study (Starkweather et al., 2013) which found no differences between the low and high symptom subgroups. The inconsistent findings between these studies may be related to differences in the categorization of low versus high symptom classes, the age of the study group, and/or the measurement of both EV and soluble cytokines. While increases in GM-CSF were found in patients with chronic fatigue syndrome (Montoya et al., 2017), depression (Schmidt et al., 2014), and chronic insomnia disorder (Xia et al., 2021), we hypothesize that increased GM-CSF may be related to a more pronounced pro-inflammatory response associated with a higher symptom burden.

In addition, EV IL-2 was elevated in our older cohort with the higher symptom burden compared to the younger cohort. IL-2 (i.e., T-cell growth factor) is responsible for regulatory T cell (T regs) maintenance and function, T cell proliferation and differentiation, and natural killer (NK) cell growth (Spolski et al., 2018). Higher levels of IL-2 were found in post-viral prolonged fatigue (Garcia et al., 2014) and increased T cell activation and production of soluble IL-2 receptors was higher in depressed individuals (Maes, 2011). Similar to GM-CSF, these differences may be related to higher symptom burden rather than the aging process. In terms of findings in aging healthy adults, a review of the literature (Rea et al., 2018) found that soluble IL-2 production decreased with age. More research is needed to evaluate our findings of elevated EV IL-2 associated with age in patients diagnosed with breast cancer.

In the younger cohort of women with a higher symptom burden, lower levels of soluble MIG remained marginally significant (*p* = 0.053) after adjusting for significant covariates. In contrast, in the older cohort, no differences in MIG levels were found between the symptom classes. MIG is a pro-inflammatory CXC chemokine family member induced by IFN-y that has chemotactic properties and targets T lymphocyte proliferation (Whiting et al., 2004). Higher levels of MIG were associated with the breast cancer development (Ding et al., 2016). We were unable to identify any studies evaluating for an association of MIG with symptom classes in breast cancer patients. In terms of the general population, MIG levels were observed to increase with age in healthy adults (Shurin et al., 2007). It is plausible that our finding, albeit marginally significant, is related to the progression of breast cancer itself rather than to differences in age or symptom classes.

To further investigate the relationships between symptom severity and cytokine levels, we performed bivariate correlation analyses for individual symptoms within each age cohort and symptom class. Several significant positive associations were identified. In terms of depressive symptoms, positive associations were found with three soluble pro-inflammatory cytokines (GRO-α, IL-1β, IL-2) in the older cohort in the High Fatigue/Low Pain class. This finding is consistent with previous studies that found that higher levels of depressive symptoms were associated with higher levels of IL-2, IL-1β, and GRO-α (Thomas et al., 2005; Chai et al., 2019; Ho et al., 2021).

In contrast, in the younger cohort in the High Fatigue/Low Pain class, higher levels of depressive symptoms were associated with lower levels of several EV (IL-22, M-CSF, MIG) and soluble (MIP-3α, TGF-β) cytokines. In addition, in the younger cohort in the All Low class, higher levels of depressive symptoms were associated with higher levels of the anti-inflammatory cytokine, IL-10. Similarly, a recent study found a negative correlation between the pro-inflammatory cytokine, IL-1β, and depressive symptoms in patients with breast cancer with a mean age of 45 years (Li et al., 2021). In another study, higher levels of depressive symptoms were associated with higher levels of IL-1β, and TNF-α post-surgery in breast cancer patients with a mean age of 50 years (Bouchard et al., 2016). While the findings in the literature are inconsistent, previous studies (Bouchard et al., 2016; Li et al., 2021) included patients with a wide age range and did not dichotomize the sample into older and younger cohorts. Our findings suggest a pro-inflammatory role in depressive symptoms among the older cohort compared to the younger cohort that is likely related to aging and cancer immune modulations.

Congruent with previous reports of the positive associations between fatigue severity and pro-inflammatory cytokines (Feng et al., 2017; Paulsen et al., 2017), we found higher fatigue to be associated with higher levels of IL-1β in the older cohort and IP-10, MIP-3β in the younger cohort of patients in High Fatigue/Low Pain class. While no positive associations were found in the older cohort for sleep disturbances in either symptom class, we observed higher levels of EV TRAIL to be associated with greater sleep disturbances in High Fatigue/Low Pain class of the younger cohort. The lack of positive association in the older cohort may be related to its smaller sample size compared to the sample size of the younger cohort.

While we did not compare concentrations of EVs and their associated cytokines between age cohorts irrespective of symptom classes, in another study, EV concentrations from plasma decreased with age, had increased internalization of EVs by B cells, and had increased Major Histocompatibility Complex-II expression on monocytes in older compared to younger adults (Eitan et al., 2017). Similarly, in a murine model (Alibhai FJ. et al., 2020) EV concentration and cargo differed between older and younger mice. In our study, we found variability in cytokine concentrations between EV and soluble fractions which suggests that some cytokines were more enriched in association with EVs. We hypothesize that this variability may be related to the distribution of soluble versus EV cytokines within the biological system of plasma, and immune system modulations within each symptom class. While, in this report, we determined total concentrations of EV cytokines using a lysis buffer, a previous report showed that some cytokine types may be preferentially encapsulated in EVs depending on the cell type releasing them and the biological system involved (Fitzgerald et al., 2018a). Thus, because EV encapsulated cytokines may not be measured by typical immune assays, the inclusion of this fraction of cytokines may provide additional insights into biology of symptoms. It is important to note that EV cargo can differ in different stages of breast cancer progression and can play a role in tumor microenvironment and cancer proliferation (Brena et al., 2022). While we did not investigate the latent classes and age cohort separately for each individual tumor stage or estrogen and progesterone receptor statuses (negative, positive), we controlled for significant clinical covariates (Table 2). We propose that future studies should enroll larger cohorts to allow for analysis of EV-associated biomarkers in each breast cancer subtypes for further validation of our preliminary results.

This study has several limitations. First, the relatively small sample sizes may have biased our results. Next, variability in times and dates of plasma collection does not account for circadian variability and storage time. Third, because of the relatively small sample sizes and exploratory nature of this study, we did not perform corrections for multiple comparisons. Fourth, because this analysis was cross-sectional, an evaluation of longitudinal changes in EVs warrants additional investigation. Lastly, while we selected the ExoQuick method for EV extraction to be able to analyze the entire EV population and because our starting sample volumes were below 500 μl, using ExoQuick yields lower purity for EV isolated fractions compared to ultracentrifugation (UC) and size exclusion chromatography (SEC). Specifically, when compared to SEC (i.e., qEV70 columns from IZON), ExoQuick shows lower purity, higher protein/lipoprotein contamination, but higher total EV yield (Pang et al., 2020). Similarly, while the UC compared to ExoQuick yields higher purity, it may result in loss of smaller subpopulations of EVs such as exosomes (Helwa et al., 2017). Future studies comparing UC, SEC, and ExoQuick in cytokine analysis will be informative to ensure these preliminary results are reproducible using other EV isolation methods.

Despite these limitations, one of the strengths of this analysis is that the sample was homogeneous in terms of cancer type and recruitment prior to breast cancer surgery. This study is novel in its examination of EVs with distinct pre-specified symptom profiles and demonstrating differences in EV and soluble cytokine expressions based on these profiles. In addition, while initial findings suggest a higher symptom burden may be related to inflammation, the exact immune pathways appear to vary between older and younger cohorts, perhaps relating to the additive effects of aging and cancer. Further exploration of these results is warranted using larger sample sizes, a priori calculated effect sizes, and longitudinal designs.

These findings, albeit preliminary, offer new avenues for the use of EVs as tools for liquid biopsy and may increase our understanding of the mechanisms that underlie the symptom experiences of older and younger oncology patients in larger cohorts over the cancer treatment trajectory. While the study raises more questions, it advances opportunities for more scientific endeavors. Future studies should incorporate longitudinal study designs, larger sample sizes that accommodate for multiple comparison corrections and other EV associated biomarkers (i.e., cytokines, miRNA) to further elucidate biologic mechanisms of age-related differences in symptom phenotypes.

## Data Availability

The original contributions presented in the study are included in the article/Supplementary Material, further inquiries can be directed to the corresponding author.
