# Supplementary material for: Differences in Circulating Extracellular Vesicle and Soluble Cytokines in Older Versus Younger Breast Cancer Patients With Distinct Symptom Profiles
Source: Front Genet. 2022 Apr 25;13:869044. doi: 10.3389/fgene.2022.869044 (PMC9081604; doi:10.3389/fgene.2022.869044)
Supplement: Supplementary file 1 [file DataSheet1.docx]

Supplementary Material

# Supplementary Methods:

## Flow cytometry verification of EVs

EV-depleted and EV-enriched fractions from four patients were characterized by flow cytometry. Fractions were labelled with a lipophilic dye, Cell Mask Deep Red (CMDR, 5 μg/ml, Thermo Fisher) to label EVs as well as any other lipoproteins. EVs were verified by the presence of tetraspanins by labelling with mouse-anti-human CD9-BV421, CD63-BV786 and CD81-BV605 (BD Bioscience). Samples were interrogated for non-EV markers by staining with mouse anti-human apolipoprotein A1 followed by goat anti-mouse IgG FITC and rabbit anti-human apolipoprotein B antibodies followed by a goat anti-rabbit IgG PE (Thermo Fisher). Finally, fractions were stained for a platelet marker by staining with mouse anti-human CD31-BV605 (BD Bioscience). Samples were acquired on a BD Symphony flow cytometer, as previously described (Mercurio et al., 2021) with forward scatter and side scatter in log mode and thresholds set on CMDR events.

## Characterization of size and concentrations of EVs

EV samples from 30 participants were characterized for size and concentration through microfluidic resistive pulse sensing using the nCS1^TM^ particle analyzer (Spectradyne, Torrance, CA). Samples were serially diluted to 1:100 in 2% Tween 20 and 1X PBS solution filtered at 0.2 μm. In total, 5 μL of diluted samples were placed in a C400 microfluidic cartridge to generate EV concentrations (EV/ml) and EV diameter size (nm). Background subtraction was used to eliminate false positives from electrical noise as visualized in the scatter plots. A polygon filter was applied to highlight the area of “included events” which visually separated the electrical noise from the particle events. After the filtering, a concentration spectral density (CSD) with a generated histogram and Gaussian distribution curve was used to estimate the mean size and diameter of each EV particle.

# Supplementary Results

## Flow cytometry characterization of isolated EVs.


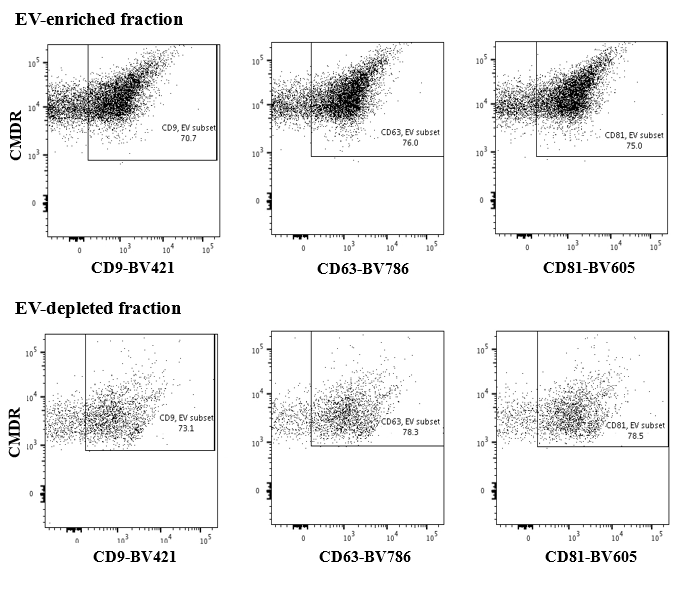


**Supplementary Figure 1. Flow cytometry characterization of EV markers.** EV-depleted and EV-enriched fractions collected by ExoQuick from platelet poor plasma were labelled with Cell Mask Deep Red (CMDR) and stained with mouse-anti-human CD9-BV421, CD63-BV786, and CD81-BV605 to identify EV-specific tetraspanin proteins. Shown is one representative sample.


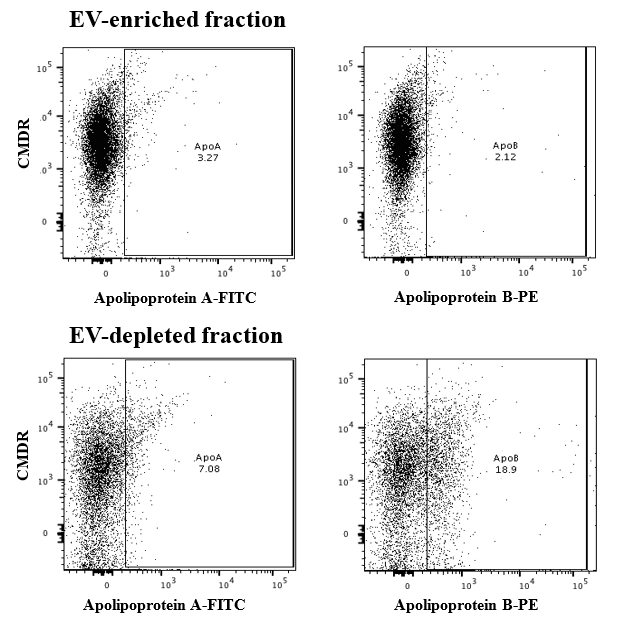


**Supplementary Figure 2. Flow cytometry characterization of non-EV markers.** EV-depleted and EV-enriched fractions collected by ExoQuick from platelet poor plasma were labelled with Cell Mask Deep Red (CMDR) and were inspected for non-EV markers by staining with mouse-anti-human anti-apolipoprotein A followed by goat-anti-mouse IgG-FITC and rabbit-anti-human lipoprotein B followed by goat anti-rabbit IgG-PE. Shown is one representative sample.


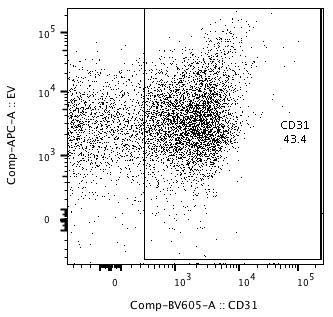

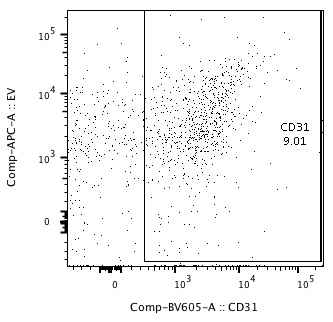


**EV-enriched fraction**

**EV-depleted fraction**

**CD31-BV605**

**CMDR**

**CD31-BV605**

**Supplementary Figure 3. Flow cytometry characterization of platelet markers.** EV-depleted and EV-enriched fractions collected by ExoQuick from platelet poor plasma were labelled with Cell Mask Deep Red (CMDR) and were inspected for platelet markers by staining with mouse anti-human CD31-BV605. Shown is one representative sample.

## Representative scatter plot and histogram using nCS1^TM^ particle analyzer.


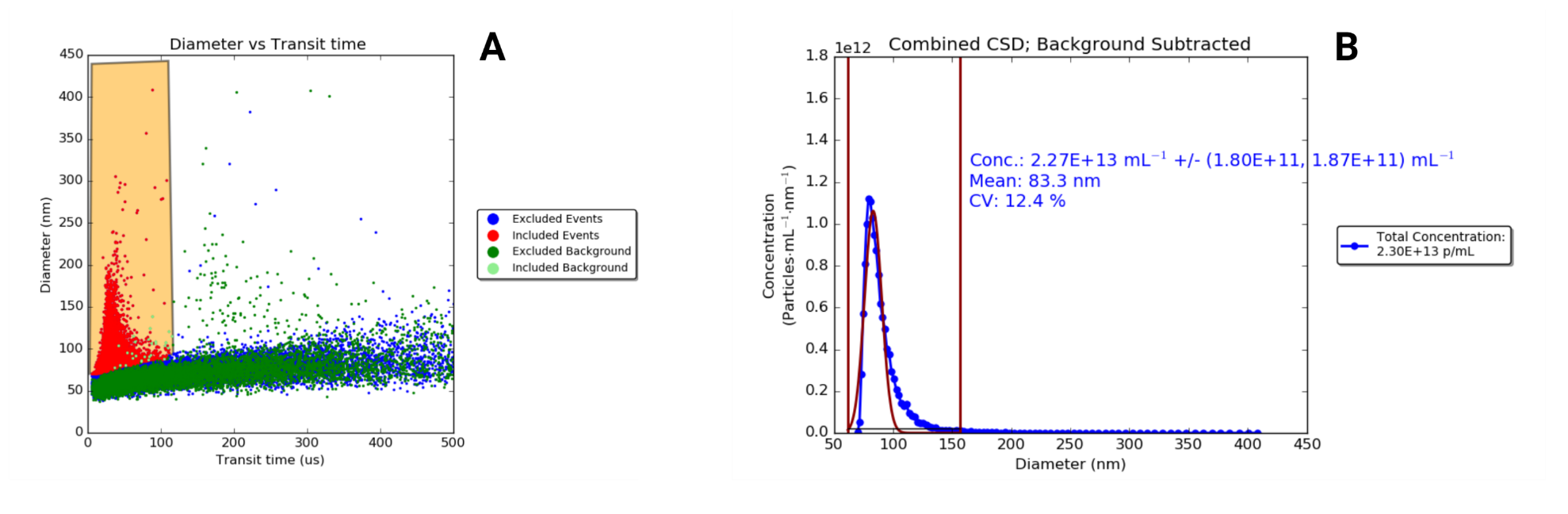


**Supplementary Figure 4**. Representative output from nCS1^TM^ particle analyzer from one of the samples. **A**) Representative Scatter plot of particle diameter (size) vs. transit time generated from a polygon filter demonstrates “included events” or true particle detection events (red) and false positive excluded events (dark green and blue). **B**) Representative concentration spectral density (CSD) and histogram obtained to quantify EV particle size distributions and concentrations with a fitted Gaussian curve.

Supplementary Table 1. Lower Limit of Detection (LLOD) for Each Assay

| **Assay** | **EV Assay LLOD** | **Soluble Assay LLOD** |
| --- | --- | --- |
| **IL-1α** | 0.04 | 0.040 |
| **IL-1β** | 0.065 | 0.07 |
| **IL-2** | 0.395 | 3.14 |
| **IL-4** | 0.17 | 0.38 |
| **IL-6** | 0.04 | 0.14 |
| **IL-7** | 0.045 | 0.18 |
| **IL-8** | 0.035 | 0.03 |
| **IL-10** | 0.605 | 0.58 |
| **IL-12 p70** | 0.06 | 0.02 |
| **IL-15** | 0.07 | 0.12 |
| **IL-16** | 0.475 | 6.90 |
| **IL-17** | 0.02 | 0.06 |
| **IL-18** | 0.04 | 0.01 |
| **IL-22** | 16.965 | 30.91 |
| **GM-CSF** | 0.08 | 2.40 |
| **GRO-α** | 7.62 | 1.63 |
| **IFN-γ** | 0.04 | 0.20 |
| **IP-10** | 2.665 | 16.89 |
| **ITAC** | 4.52 | 2.74 |
| **M-CSF** | 0.1 | 0.04 |
| **MCP-1** | 0.245 | 0.20 |
| **MIG** | 46.595 | 78.98 |
| **MIP-1α** | 0.015 | 0.04 |
| **MIP-1β** | 0.05 | 0.25 |
| **MIP-3α** | 8.125 | 1.40 |
| **TGF-β** | 2.05 | 1.46 |
| **TNF-α** | 0.065 | 0.13 |
| **IL-1RA** | 1.31 | 0.03 |
| **MIP-3β** | 0.21 | 0.52 |
| **PD-L1** | 0.55 | 1.45 |
| **TRAIL** | 2.675 | 4.83 |
| **VEGF** | 1.43 | 1.15 |

Cytokines were measured across 9 plates and the minimum lower limit of detection (LLOD)/2 was used to replace values marked as “<OOR” using Beal’s approach (Senn et al., 2012). Values are expressed in pg/ml.

**Abbreviations:** α = alpha, β = beta , EV = extracellular vesicle, GM-CSF = granulocyte-macrophage colony-stimulating factor, GRO-α = growth-regulated alpha, IFN-γ = interferon-γ, IP-10 = interferon-γ-induced protein, IL = interleukin, ITAC = interferon-inducible T-cell alpha chemoattractant, LLOD = lower limit of detection, M-CSF = macrophage colony-stimulating factor, MCP-1 = monocyte chemoattractant protein-1, MIG = monokine induced by IFN-γ, MIP-1α = macrophage inflammatory protein-1α, OOR = out of range, PD-L1 = programmed death-ligand 1, RA = receptor antagonist, TGF-β = transforming growth factor beta, TNF-α = tumor necrosis factor-α, TRAIL = TNF- related apoptosis-inducing ligand, VEGF = vascular endothelial growth factor.

## Supplementary Table 2. Normality Statistics

|  | N Stat. | Mean | SD | Skew. Stat. | Skew. Std. Error | Kurt. Stat. | Kurt. Std. Error | Skew/Std. Error | Kurt/Std. Error |
| --- | --- | --- | --- | --- | --- | --- | --- | --- | --- |
| Age ≥ 60 years | | | | | | | | | |
| Age at Enrollment | 93.00 | 68.23 | 6.47 | 1.10 | 0.25 | 0.67 | 0.50 | 4.41 | 1.35 |
| Years of Education | 93.00 | 15.03 | 2.48 | 0.00 | 0.25 | 0.04 | 0.50 | 0.01 | 0.09 |
| Number of breast biopsies in the past year | 82.00 | 1.24 | 0.53 | 2.15 | 0.27 | 3.77 | 0.53 | 8.08 | 7.17 |
| Karnofsky Performance Status Score - Enrollment | 91.00 | 97.03 | 6.24 | -2.51 | 0.25 | 6.98 | 0.50 | -9.92 | 13.96 |
| Body Mass Index | 90.00 | 27.52 | 6.38 | 1.17 | 0.25 | 1.55 | 0.50 | 4.60 | 3.08 |
| IL-1α EV | 91.00 | -0.17 | 2.89 | -0.09 | 0.25 | -0.04 | 0.50 | -0.37 | -0.07 |
| IL-1β EV | 93.00 | 0.01 | 3.03 | -0.16 | 0.25 | -1.09 | 0.50 | -0.63 | -2.20 |
| IL-2 EV | 93.00 | 1.97 | 2.84 | -0.05 | 0.25 | -1.60 | 0.50 | -0.19 | -3.23 |
| IL-4 EV | 93.00 | 2.19 | 3.42 | -0.05 | 0.25 | -0.57 | 0.50 | -0.19 | -1.15 |
| IL-6 EV | 93.00 | -0.91 | 3.19 | 0.06 | 0.25 | -1.10 | 0.50 | 0.25 | -2.22 |
| IL-7 EV | 93.00 | 0.26 | 3.51 | -0.27 | 0.25 | -1.39 | 0.50 | -1.08 | -2.81 |
| IL-8 EV | 92.00 | -0.34 | 3.28 | -0.29 | 0.25 | -1.35 | 0.50 | -1.16 | -2.71 |
| IL-10 EV | 93.00 | -0.17 | 1.88 | 3.32 | 0.25 | 9.92 | 0.50 | 13.29 | 20.04 |
| IL-12p70 EV | 92.00 | 1.84 | 4.30 | -0.06 | 0.25 | -0.92 | 0.50 | -0.24 | -1.85 |
| IL-15 EV | 93.00 | -1.93 | 2.47 | 0.97 | 0.25 | -0.09 | 0.50 | 3.88 | -0.18 |
| IL-16 EV | 93.00 | 8.41 | 1.83 | 0.25 | 0.25 | -0.82 | 0.50 | 1.00 | -1.66 |
| IL-17 EV | 93.00 | -3.87 | 2.75 | 1.52 | 0.25 | 1.93 | 0.50 | 6.10 | 3.90 |
| IL-18 EV | 93.00 | 1.14 | 3.04 | -0.80 | 0.25 | -0.04 | 0.50 | -3.19 | -0.09 |
| IL-22 EV | 93.00 | 5.66 | 2.01 | 0.58 | 0.25 | -1.53 | 0.50 | 2.32 | -3.10 |
| GM-CSF EV | 93.00 | 0.28 | 4.12 | 0.20 | 0.25 | -1.81 | 0.50 | 0.81 | -3.65 |
| GRO-α EV | 93.00 | 6.50 | 2.17 | -0.69 | 0.25 | -0.84 | 0.50 | -2.77 | -1.69 |
| IFN-γ EV | 89.00 | -0.50 | 5.33 | 0.71 | 0.26 | -1.08 | 0.51 | 2.79 | -2.14 |
| IP-10 EV | 93.00 | 9.21 | 1.82 | -1.45 | 0.25 | 6.13 | 0.50 | -5.82 | 12.37 |
| ITAC EV | 93.00 | 10.01 | 1.83 | -0.72 | 0.25 | 4.76 | 0.50 | -2.89 | 9.61 |
| M-CSF EV | 93.00 | 1.52 | 2.48 | -0.40 | 0.25 | 0.45 | 0.50 | -1.59 | 0.90 |
| MCP-1 EV | 93.00 | -0.49 | 2.81 | 1.42 | 0.25 | 0.31 | 0.50 | 5.67 | 0.63 |
| MIG EV | 93.00 | 11.31 | 1.27 | -2.95 | 0.25 | 12.45 | 0.50 | -11.79 | 25.14 |
| MIP-1α EV | 92.00 | 4.00 | 0.89 | 0.74 | 0.25 | 2.05 | 0.50 | 2.96 | 4.11 |
| MIP-1β EV | 93.00 | 4.48 | 0.82 | 0.02 | 0.25 | -0.10 | 0.50 | 0.10 | -0.19 |
| MIP-3α EV | 93.00 | 4.18 | 2.53 | 2.04 | 0.25 | 3.00 | 0.50 | 8.16 | 6.07 |
| TGF-β EV | 93.00 | 6.26 | 3.45 | -0.34 | 0.25 | -1.01 | 0.50 | -1.34 | -2.05 |
| TNF-α EV | 93.00 | 0.58 | 2.19 | -0.93 | 0.25 | 0.24 | 0.50 | -3.74 | 0.48 |
| IL-1RA EV | 93.00 | 5.57 | 3.52 | -0.24 | 0.25 | -0.89 | 0.50 | -0.96 | -1.79 |
| MIP-3β EV | 93.00 | 9.74 | 1.63 | -0.33 | 0.25 | 1.35 | 0.50 | -1.33 | 2.73 |
| PD-L1 EV | 93.00 | 2.61 | 3.13 | 0.09 | 0.25 | -1.25 | 0.50 | 0.37 | -2.52 |
| TRAIL EV | 93.00 | 6.01 | 1.22 | -1.64 | 0.25 | 4.84 | 0.50 | -6.54 | 9.77 |
| VEGF EV | 92.00 | 6.30 | 3.08 | -1.08 | 0.25 | -0.23 | 0.50 | -4.30 | -0.45 |
| IL-1α soluble | 93.00 | -0.56 | 2.37 | -0.56 | 0.25 | -0.47 | 0.50 | -2.22 | -0.94 |
| IL-1β soluble | 93.00 | 0.19 | 2.87 | -0.32 | 0.25 | -1.64 | 0.50 | -1.29 | -3.31 |
| IL-2 soluble | 93.00 | 4.18 | 2.19 | 0.21 | 0.25 | -1.51 | 0.50 | 0.85 | -3.06 |
| IL-4 soluble | 93.00 | 1.65 | 2.22 | -0.14 | 0.25 | -1.44 | 0.50 | -0.58 | -2.91 |
| IL-6 soluble | 93.00 | 1.33 | 3.26 | -0.23 | 0.25 | -1.77 | 0.50 | -0.93 | -3.57 |
| IL-7 soluble | 93.00 | 4.07 | 1.50 | -2.24 | 0.25 | 7.85 | 0.50 | -8.96 | 15.85 |
| IL-8 soluble | 93.00 | 2.39 | 2.34 | -2.02 | 0.25 | 4.09 | 0.50 | -8.08 | 8.25 |
| IL-10 soluble | 93.00 | 0.51 | 2.25 | 2.03 | 0.25 | 2.50 | 0.50 | 8.14 | 5.06 |
| IL-12p70 soluble | 93.00 | -0.26 | 4.21 | 0.04 | 0.25 | -1.17 | 0.50 | 0.16 | -2.36 |
| IL-15 soluble | 93.00 | 2.09 | 1.76 | -1.02 | 0.25 | 1.17 | 0.50 | -4.08 | 2.37 |
| IL-16 soluble | 93.00 | 11.09 | 1.47 | 0.17 | 0.25 | -0.92 | 0.50 | 0.68 | -1.86 |
| IL-17 soluble | 93.00 | -1.92 | 2.33 | 0.83 | 0.25 | -0.75 | 0.50 | 3.32 | -1.51 |
| IL-18 soluble | 93.00 | 6.96 | 1.30 | 0.29 | 0.25 | -0.08 | 0.50 | 1.15 | -0.16 |
| IL-22 soluble | 93.00 | 6.96 | 1.66 | 0.17 | 0.25 | -1.58 | 0.50 | 0.67 | -3.18 |
| GM-CSF soluble | 93.00 | 3.76 | 1.86 | 0.00 | 0.25 | -1.39 | 0.50 | -0.02 | -2.81 |
| GRO-α soluble | 93.00 | 6.64 | 2.79 | -1.22 | 0.25 | 0.19 | 0.50 | -4.89 | 0.38 |
| IFN-γ soluble | 93.00 | 0.69 | 3.62 | 0.71 | 0.25 | -1.27 | 0.50 | 2.85 | -2.56 |
| IP-10 soluble | 93.00 | 11.69 | 1.19 | 0.27 | 0.25 | 1.78 | 0.50 | 1.07 | 3.60 |
| ITAC soluble | 93.00 | 10.29 | 1.13 | 0.30 | 0.25 | 2.20 | 0.50 | 1.20 | 4.45 |
| M-CSF soluble | 93.00 | 2.73 | 1.85 | -1.39 | 0.25 | 3.81 | 0.50 | -5.56 | 7.70 |
| MCP-1 soluble | 92.00 | 6.26 | 1.77 | -3.43 | 0.25 | 13.91 | 0.50 | -13.66 | 27.94 |
| MIG soluble | 93.00 | 12.69 | 0.77 | 0.18 | 0.25 | 0.90 | 0.50 | 0.70 | 1.82 |
| MIP-1α soluble | 93.00 | 4.63 | 0.87 | 0.77 | 0.25 | 1.85 | 0.50 | 3.06 | 3.74 |
| MIP-1β soluble | 91.00 | 5.63 | 0.66 | -0.59 | 0.25 | 0.54 | 0.50 | -2.35 | 1.07 |
| MIP-3α soluble | 93.00 | 2.31 | 2.77 | 1.45 | 0.25 | 0.33 | 0.50 | 5.79 | 0.68 |
| TGF-β soluble | 93.00 | 4.35 | 3.33 | 0.07 | 0.25 | -1.67 | 0.50 | 0.29 | -3.37 |
| TNF-α soluble | 93.00 | 1.92 | 1.83 | -1.44 | 0.25 | 1.81 | 0.50 | -5.77 | 3.66 |
| IL-1RA soluble | 93.00 | 4.84 | 4.70 | -1.40 | 0.25 | 0.40 | 0.50 | -5.61 | 0.81 |
| MIP-3β soluble | 93.00 | 7.24 | 3.34 | -1.41 | 0.25 | 1.14 | 0.50 | -5.64 | 2.30 |
| PD-L1 soluble | 93.00 | 4.48 | 2.07 | -0.73 | 0.25 | -0.56 | 0.50 | -2.91 | -1.14 |
| TRAIL soluble | 93.00 | 8.04 | 0.96 | -1.88 | 0.25 | 10.09 | 0.50 | -7.52 | 20.38 |
| VEGF soluble | 93.00 | 6.60 | 3.50 | -0.94 | 0.25 | -0.62 | 0.50 | -3.77 | -1.25 |
| Age <60 years | | | | | | | | | |
| Age at Enrollment | 197.00 | 48.88 | 7.29 | -0.71 | 0.17 | 0.30 | 0.34 | -4.10 | 0.86 |
| Years of Education | 195.00 | 16.18 | 2.55 | 0.11 | 0.17 | -0.21 | 0.35 | 0.64 | -0.60 |
| Number of breast biopsies | 189.00 | 1.65 | 0.91 | 1.54 | 0.18 | 2.00 | 0.35 | 8.69 | 5.69 |
| Karnofsky Performance Status Score | 194.00 | 92.53 | 10.79 | -1.79 | 0.17 | 3.66 | 0.35 | -10.28 | 10.55 |
| Body Mass Index | 195.00 | 26.38 | 6.25 | 1.84 | 0.17 | 5.87 | 0.35 | 10.54 | 16.95 |
| IL-1α EV | 195.00 | -0.50 | 2.72 | -0.28 | 0.17 | -0.71 | 0.35 | -1.62 | -2.05 |
| IL-1β EV | 197.00 | 0.34 | 2.88 | -0.42 | 0.17 | -1.10 | 0.34 | -2.44 | -3.19 |
| IL-2 EV | 197.00 | 2.13 | 2.97 | 0.03 | 0.17 | -1.41 | 0.34 | 0.15 | -4.10 |
| IL-4 EV | 196.00 | 1.87 | 3.11 | -0.08 | 0.17 | -0.92 | 0.35 | -0.47 | -2.66 |
| IL-6 EV | 197.00 | -1.30 | 3.01 | 0.05 | 0.17 | -1.58 | 0.34 | 0.30 | -4.57 |
| IL-7 EV | 196.00 | 0.31 | 3.57 | -0.32 | 0.17 | -1.44 | 0.35 | -1.85 | -4.18 |
| IL-8 EV | 197.00 | -0.69 | 3.14 | -0.22 | 0.17 | -1.37 | 0.34 | -1.27 | -3.98 |
| IL-10 EV | 197.00 | -0.35 | 1.51 | 3.99 | 0.17 | 14.86 | 0.34 | 23.02 | 43.11 |
| IL-12p70 EV | 197.00 | 2.50 | 4.20 | -0.18 | 0.17 | -0.79 | 0.34 | -1.04 | -2.30 |
| IL-15 EV | 196.00 | -1.54 | 2.54 | 0.50 | 0.17 | -1.21 | 0.35 | 2.87 | -3.50 |
| IL-16 EV | 197.00 | 8.03 | 1.59 | 0.23 | 0.17 | -0.01 | 0.34 | 1.35 | -0.03 |
| IL-17 EV | 196.00 | -3.97 | 2.63 | 1.27 | 0.17 | 0.48 | 0.35 | 7.32 | 1.39 |
| IL-18 EV | 197.00 | 0.46 | 2.95 | -0.68 | 0.17 | -0.65 | 0.34 | -3.94 | -1.90 |
| IL-22 EV | 197.00 | 5.75 | 2.03 | 0.48 | 0.17 | -1.57 | 0.34 | 2.77 | -4.55 |
| GM-CSF EV | 197.00 | 0.37 | 4.01 | 0.12 | 0.17 | -1.77 | 0.34 | 0.67 | -5.13 |
| GRO-α EV | 197.00 | 6.68 | 2.08 | -0.75 | 0.17 | -0.49 | 0.34 | -4.36 | -1.43 |
| IFN-γ EV | 196.00 | -1.13 | 4.93 | 0.84 | 0.17 | -0.98 | 0.35 | 4.86 | -2.84 |
| IP-10 EV | 197.00 | 8.57 | 2.14 | -1.58 | 0.17 | 4.46 | 0.34 | -9.09 | 12.95 |
| ITAC EV | 197.00 | 9.79 | 1.22 | -0.94 | 0.17 | 7.84 | 0.34 | -5.43 | 22.73 |
| M-CSF EV | 196.00 | 1.67 | 2.03 | -0.89 | 0.17 | 0.92 | 0.35 | -5.14 | 2.67 |
| MCP-1 EV | 197.00 | -0.91 | 2.40 | 1.76 | 0.17 | 1.31 | 0.34 | 10.19 | 3.79 |
| MIG EV | 197.00 | 11.28 | 0.93 | -2.60 | 0.17 | 13.28 | 0.34 | -14.99 | 38.53 |
| MIP-1α EV | 197.00 | 3.85 | 0.87 | 0.21 | 0.17 | 0.41 | 0.34 | 1.19 | 1.17 |
| MIP-1β EV | 197.00 | 4.22 | 0.81 | 0.17 | 0.17 | 0.33 | 0.34 | 0.99 | 0.95 |
| MIP-3α EV | 197.00 | 3.89 | 1.96 | 2.02 | 0.17 | 2.63 | 0.34 | 11.65 | 7.64 |
| TGF-β EV | 197.00 | 6.14 | 3.63 | -0.31 | 0.17 | -1.21 | 0.34 | -1.77 | -3.51 |
| TNF-α EV | 197.00 | 0.48 | 2.31 | -0.69 | 0.17 | -0.26 | 0.34 | -3.99 | -0.76 |
| IL-1RA EV | 197.00 | 5.36 | 3.30 | -0.39 | 0.17 | -0.99 | 0.34 | -2.25 | -2.88 |
| MIP-3β EV | 197.00 | 9.61 | 2.34 | -2.58 | 0.17 | 11.45 | 0.34 | -14.92 | 33.21 |
| PD-L1 EV | 197.00 | 2.53 | 3.08 | 0.22 | 0.17 | -0.83 | 0.34 | 1.26 | -2.41 |
| TRAIL EV | 196.00 | 6.03 | 1.26 | -1.51 | 0.17 | 4.48 | 0.35 | -8.67 | 12.95 |
| VEGF EV | 197.00 | 5.81 | 3.05 | -0.83 | 0.17 | -0.71 | 0.34 | -4.80 | -2.06 |
| IL-1α soluble | 197.00 | -0.66 | 2.18 | -0.84 | 0.17 | -0.58 | 0.34 | -4.87 | -1.68 |
| IL-1β soluble | 197.00 | 0.50 | 2.62 | -0.57 | 0.17 | -1.11 | 0.34 | -3.29 | -3.21 |
| IL-2 soluble | 197.00 | 4.43 | 2.21 | 0.05 | 0.17 | -1.52 | 0.34 | 0.26 | -4.41 |
| IL-4 soluble | 197.00 | 1.11 | 2.16 | 0.18 | 0.17 | -1.65 | 0.34 | 1.02 | -4.77 |
| IL-6 soluble | 197.00 | 0.65 | 3.26 | 0.18 | 0.17 | -1.77 | 0.34 | 1.04 | -5.13 |
| IL-7 soluble | 197.00 | 3.80 | 1.92 | -1.93 | 0.17 | 3.71 | 0.34 | -11.14 | 10.76 |
| IL-8 soluble | 197.00 | 1.96 | 2.63 | -1.52 | 0.17 | 1.77 | 0.34 | -8.78 | 5.13 |
| IL-10 soluble | 197.00 | 0.29 | 2.03 | 2.53 | 0.17 | 4.86 | 0.34 | 14.61 | 14.11 |
| IL-12p70 soluble | 196.00 | -0.64 | 4.75 | 0.36 | 0.17 | -1.28 | 0.35 | 2.06 | -3.72 |
| IL-15 soluble | 197.00 | 2.34 | 1.86 | -1.16 | 0.17 | 1.50 | 0.34 | -6.71 | 4.36 |
| IL-16 soluble | 196.00 | 10.90 | 1.25 | 0.45 | 0.17 | -0.50 | 0.35 | 2.59 | -1.45 |
| IL-17 soluble | 197.00 | -2.23 | 2.17 | 0.93 | 0.17 | -0.80 | 0.34 | 5.37 | -2.33 |
| IL-18 soluble | 197.00 | 6.78 | 1.14 | 0.35 | 0.17 | -0.11 | 0.34 | 2.02 | -0.31 |
| IL-22 soluble | 197.00 | 7.00 | 1.66 | 0.17 | 0.17 | -1.52 | 0.34 | 0.97 | -4.40 |
| GM-CSF soluble | 197.00 | 3.68 | 1.93 | 0.17 | 0.17 | -1.39 | 0.34 | 0.99 | -4.03 |
| GRO-α soluble | 197.00 | 6.37 | 3.05 | -0.96 | 0.17 | -0.62 | 0.34 | -5.56 | -1.79 |
| IFN-γ soluble | 197.00 | 0.53 | 3.54 | 0.78 | 0.17 | -1.18 | 0.34 | 4.48 | -3.42 |
| IP-10 soluble | 197.00 | 11.15 | 1.28 | -1.68 | 0.17 | 7.60 | 0.34 | -9.69 | 22.04 |
| ITAC soluble | 197.00 | 10.13 | 0.84 | -0.40 | 0.17 | -0.46 | 0.34 | -2.29 | -1.35 |
| M-CSF soluble | 197.00 | 2.79 | 1.51 | -2.06 | 0.17 | 8.38 | 0.34 | -11.88 | 24.31 |
| MCP-1 soluble | 197.00 | 6.14 | 1.54 | -3.08 | 0.17 | 14.21 | 0.34 | -17.78 | 41.22 |
| MIG soluble | 197.00 | 12.31 | 0.92 | -2.37 | 0.17 | 14.01 | 0.34 | -13.71 | 40.63 |
| MIP-1α soluble | 196.00 | 4.48 | 0.87 | 0.62 | 0.17 | 0.83 | 0.35 | 3.54 | 2.39 |
| MIP-1β soluble | 197.00 | 5.56 | 0.65 | -0.09 | 0.17 | 0.21 | 0.34 | -0.51 | 0.62 |
| MIP-3α soluble | 197.00 | 2.48 | 2.79 | 1.21 | 0.17 | -0.28 | 0.34 | 7.00 | -0.81 |
| TGF-β soluble | 197.00 | 4.25 | 3.57 | 0.28 | 0.17 | -1.62 | 0.34 | 1.62 | -4.69 |
| TNF-α soluble | 197.00 | 1.56 | 2.26 | -0.93 | 0.17 | -0.33 | 0.34 | -5.36 | -0.95 |
| IL-1RA soluble | 195.00 | 3.93 | 5.06 | -1.00 | 0.17 | -0.73 | 0.35 | -5.74 | -2.10 |
| MIP-3β soluble | 197.00 | 6.98 | 3.47 | -1.29 | 0.17 | 0.68 | 0.34 | -7.43 | 1.97 |
| PD-L1 soluble | 197.00 | 4.40 | 2.00 | -0.80 | 0.17 | -0.70 | 0.34 | -4.60 | -2.02 |
| TRAIL soluble | 197.00 | 8.10 | 0.87 | -1.12 | 0.17 | 7.60 | 0.34 | -6.44 | 22.06 |
| VEGF soluble | 197.00 | 6.23 | 3.27 | -0.89 | 0.17 | -0.63 | 0.34 | -5.12 | -1.81 |

**Abbreviations**: α = alpha, β = beta, EV = extracellular vesicles, GM-CSF = granulocyte-macrophage colony-stimulating factor, GRO-α = growth-regulated alpha, IFN-γ = interferon-γ, IP-10 = interferon-γ-induced protein, IL = interleukin, ITAC = interferon-inducible T-cell alpha chemoattractant, Kurt = kurtosis statistics, M-CSF = macrophage colony-stimulating factor, MCP-1 = monocyte chemoattractant protein-1, MIG = monokine induced by IFN-γ, MIP-1α = macrophage inflammatory protein-1α, PD-L1 = programmed death-ligand 1, RA = receptor antagonist, SD = standard deviation, Std. = standard, Skew = skewness statistics, Stat = statistics, TGF-β = transforming growth factor beta, TNF-α = tumor necrosis factor-α, TRAIL = TNF- related apoptosis-inducing ligand, VEGF = vascular endothelial growth factor.

## Supplementary Table 3. Bivariate Correlation Analyses in the Older Cohort (age ≥60 years of age)

|  |  | **High Fatigue/Low Pain class** | | | | | | | | | **All Low class** | | | | | | | | |
| --- | --- | --- | --- | --- | --- | --- | --- | --- | --- | --- | --- | --- | --- | --- | --- | --- | --- | --- | --- |
| **Assay** | **Measure** | **CSD** | | | **GSDS** | | | **LFS** | | | **CSD** | | | **GSDS** | | | **LFS** | | |
|  |  | **r** | **p** | **n** | **r** | **p** | **n** | **r** | **p** | **n** | **r** | **p** | **n** | **r** | **p** | **n** | **r** | **p** | **n** |
| GM-CSF | **EV** | 0.47 | 0.12 | 14 | 0.04 | 0.89 | 15 | 0.17 | 0.60 | 14 | 0.06 | 0.76 | 29 | 0.14 | 0.50 | 29 | 0.29 | 0.15 | 28 |
|  | **Sol** | 0.38 | 0.25 | 13 | 0.22 | 0.50 | 14 | 0.38 | 0.23 | 14 | -0.10 | 0.52 | 43 | -0.12 | 0.45 | 42 | -0.15 | 0.37 | 41 |
| GRO-α | **EV** | 0.29 | 0.30 | 17 | -0.14 | 0.61 | 18 | -0.03 | 0.92 | 17 | 0.02 | 0.87 | 50 | 0.08 | 0.61 | 49 | -0.01 | 0.96 | 48 |
|  | **Sol** | 0.56 | 0.04 | 16 | 0.25 | 0.38 | 17 | 0.44 | 0.11 | 16 | 0.02 | 0.91 | 55 | -0.26 | 0.07 | 54 | -0.13 | 0.37 | 53 |
| IFN-γ | **EV** | -0.16 | 0.80 | 7 | -0.55 | 0.26 | 8 | 0.09 | 0.87 | 8 | 0.11 | 0.61 | 25 | -0.03 | 0.89 | 25 | -0.07 | 0.75 | 25 |
|  | **Sol** | 0.67 | 0.53 | 5 | -0.99 | 0.01 | 6 | -0.68 | 0.32 | 6 | -0.11 | 0.59 | 28 | -0.37 | 0.06 | 28 | 0.16 | 0.45 | 26 |
| IL-10 | **EV** | -0.73 | ND | 3 | -0.70 | ND | 3 | -0.70 | ND | 3 | 0.92 | 0.26 | 5 | 0.79 | 0.42 | 5 | 0.92 | 0.26 | 5 |
|  | **Sol** | -0.24 | ND | 3 | 0.07 | ND | 3 | -1.00 | ND | 3 | -0.25 | 0.48 | 12 | -0.16 | 0.67 | 12 | 0.03 | 0.93 | 12 |
| IL-12p70 | **EV** | 0.25 | 0.41 | 15 | -0.09 | 0.77 | 16 | 0.20 | 0.52 | 15 | 0.12 | 0.44 | 47 | 0.07 | 0.66 | 46 | -0.02 | 0.92 | 46 |
|  | **Sol** | 0.13 | 0.67 | 15 | 0.10 | 0.73 | 16 | 0.49 | 0.09 | 15 | -0.04 | 0.82 | 43 | 0.01 | 0.96 | 42 | -0.27 | 0.09 | 41 |
| IL-15 | **EV** | -0.42 | 0.49 | 7 | -0.24 | 0.64 | 8 | -0.04 | 0.95 | 7 | 0.01 | 0.95 | 31 | -0.06 | 0.77 | 31 | 0.12 | 0.53 | 31 |
|  | **Sol** | 0.31 | 0.24 | 18 | -0.17 | 0.53 | 18 | -0.13 | 0.64 | 17 | 0.20 | 0.12 | 64 | -0.10 | 0.44 | 63 | -0.12 | 0.36 | 62 |
| IL-16 | **EV** | 0.48 | 0.06 | 18 | 0.20 | 0.45 | 19 | 0.13 | 0.63 | 18 | -0.07 | 0.61 | 67 | 0.04 | 0.75 | 66 | 0.06 | 0.65 | 65 |
|  | **Sol** | 0.42 | 0.10 | 18 | 0.09 | 0.75 | 19 | 0.24 | 0.38 | 18 | -0.03 | 0.84 | 67 | -0.09 | 0.50 | 66 | -0.07 | 0.58 | 65 |
| IL-17 | **EV** | -0.94 | 0.06 | 6 | -0.99 | 0.02 | 6 | -0.76 | 0.25 | 6 | 0.10 | 0.66 | 26 | -0.10 | 0.64 | 26 | -0.13 | 0.55 | 26 |
|  | **Sol** | -0.26 | 0.74 | 6 | -0.42 | 0.48 | 7 | -0.38 | 0.53 | 7 | -0.08 | 0.66 | 33 | 0.23 | 0.22 | 32 | -0.10 | 0.61 | 32 |
| IL-18 | **EV** | 0.00 | 0.99 | 15 | -0.03 | 0.91 | 16 | 0.15 | 0.62 | 15 | 0.07 | 0.62 | 55 | 0.06 | 0.70 | 54 | 0.23 | 0.10 | 54 |
|  | **Sol** | 0.18 | 0.50 | 18 | -0.31 | 0.23 | 19 | -0.07 | 0.79 | 18 | 0.14 | 0.26 | 67 | -0.14 | 0.27 | 66 | -0.12 | 0.37 | 65 |
| IL-1RA | **EV** | 0.30 | 0.32 | 15 | -0.01 | 0.99 | 16 | 0.04 | 0.89 | 15 | -0.04 | 0.79 | 51 | -0.13 | 0.38 | 50 | 0.00 | 0.98 | 50 |
|  | **Sol** | 0.00 | 1.00 | 15 | -0.28 | 0.34 | 16 | -0.01 | 0.98 | 16 | 0.07 | 0.65 | 54 | 0.14 | 0.32 | 53 | 0.02 | 0.91 | 52 |
| IL-1α | **EV** | 0.35 | 0.30 | 13 | -0.07 | 0.84 | 14 | -0.29 | 0.36 | 14 | -0.13 | 0.36 | 54 | -0.02 | 0.91 | 53 | -0.01 | 0.95 | 53 |
|  | **Sol** | 0.48 | 0.08 | 16 | 0.24 | 0.40 | 17 | 0.06 | 0.84 | 16 | -0.05 | 0.72 | 50 | 0.01 | 0.94 | 49 | 0.03 | 0.83 | 49 |
| IL-1β | **EV** | 0.15 | 0.64 | 14 | -0.33 | 0.27 | 15 | -0.09 | 0.78 | 14 | 0.06 | 0.71 | 47 | -0.11 | 0.48 | 46 | -0.23 | 0.13 | 46 |
|  | **Sol** | 0.55 | 0.05 | 15 | 0.39 | 0.17 | 16 | 0.63 | 0.02 | 15 | 0.03 | 0.83 | 45 | -0.02 | 0.91 | 44 | -0.05 | 0.75 | 45 |
| IL-2 | **EV** | -0.56 | 0.05 | 15 | -0.41 | 0.15 | 16 | -0.18 | 0.56 | 15 | 0.10 | 0.55 | 39 | -0.09 | 0.62 | 38 | -0.10 | 0.55 | 38 |
|  | **Sol** | 0.74 | 0.04 | 10 | -0.08 | 0.85 | 11 | 0.08 | 0.85 | 10 | 0.04 | 0.85 | 36 | -0.05 | 0.79 | 35 | -0.14 | 0.45 | 34 |
| IL-22 | **EV** | -0.56 | 0.25 | 8 | -0.80 | 0.03 | 9 | -0.37 | 0.41 | 9 | 0.22 | 0.31 | 25 | 0.08 | 0.71 | 25 | 0.01 | 0.97 | 24 |
|  | **Sol** | 0.76 | 0.05 | 9 | 0.12 | 0.79 | 10 | 0.10 | 0.82 | 10 | -0.23 | 0.16 | 39 | -0.27 | 0.11 | 38 | -0.08 | 0.64 | 38 |
| IL-4 | **EV** | -0.07 | 0.84 | 14 | -0.11 | 0.73 | 15 | 0.16 | 0.60 | 15 | -0.12 | 0.44 | 48 | 0.11 | 0.48 | 47 | 0.00 | 0.99 | 47 |
|  | **Sol** | 0.26 | 0.49 | 11 | 0.26 | 0.47 | 12 | 0.55 | 0.10 | 12 | -0.14 | 0.36 | 45 | 0.07 | 0.65 | 45 | -0.03 | 0.87 | 43 |
| IL-6 | **EV** | -0.26 | 0.49 | 11 | -0.55 | 0.10 | 12 | -0.37 | 0.33 | 11 | 0.04 | 0.79 | 45 | 0.00 | 0.99 | 45 | -0.17 | 0.28 | 44 |
|  | **Sol** | 0.25 | 0.55 | 10 | -0.10 | 0.80 | 11 | 0.42 | 0.27 | 11 | -0.04 | 0.79 | 43 | 0.14 | 0.40 | 42 | -0.03 | 0.85 | 41 |
| IL-7 | **EV** | -0.50 | 0.12 | 13 | -0.31 | 0.32 | 14 | -0.31 | 0.34 | 14 | -0.10 | 0.52 | 48 | -0.09 | 0.56 | 48 | -0.33 | 0.03 | 46 |
|  | **Sol** | -0.24 | 0.39 | 17 | -0.12 | 0.66 | 18 | 0.25 | 0.37 | 17 | 0.01 | 0.96 | 65 | -0.10 | 0.43 | 64 | -0.05 | 0.69 | 63 |
| IL-8 | **EV** | -0.42 | 0.34 | 9 | -0.51 | 0.20 | 10 | -0.77 | 0.03 | 10 | 0.09 | 0.53 | 49 | 0.05 | 0.76 | 48 | -0.12 | 0.44 | 47 |
|  | **Sol** | 0.19 | 0.53 | 15 | -0.30 | 0.29 | 16 | -0.32 | 0.26 | 16 | 0.11 | 0.41 | 63 | -0.23 | 0.08 | 62 | -0.18 | 0.16 | 61 |
| IP-10 | **EV** | -0.22 | 0.42 | 17 | -0.35 | 0.18 | 18 | -0.33 | 0.22 | 17 | -0.19 | 0.14 | 66 | -0.03 | 0.84 | 65 | 0.02 | 0.88 | 64 |
|  | **Sol** | -0.07 | 0.81 | 18 | -0.36 | 0.16 | 19 | -0.55 | 0.03 | 18 | 0.08 | 0.55 | 67 | -0.07 | 0.58 | 66 | 0.01 | 0.97 | 65 |
| ITAC | **EV** | 0.14 | 0.61 | 18 | -0.17 | 0.53 | 19 | -0.04 | 0.88 | 18 | -0.02 | 0.89 | 67 | -0.03 | 0.80 | 66 | 0.08 | 0.56 | 65 |
|  | **Sol** | 0.11 | 0.69 | 18 | -0.10 | 0.71 | 19 | -0.08 | 0.77 | 18 | 0.18 | 0.14 | 67 | -0.08 | 0.55 | 66 | -0.10 | 0.45 | 65 |
| MCP-1 | **EV** | 0.04 | ND | 3 | 0.43 | ND | 4 | 0.43 | ND | 4 | -0.06 | 0.84 | 17 | 0.31 | 0.26 | 17 | 0.57 | 0.03 | 17 |
|  | **Sol** | 0.29 | 0.28 | 18 | 0.17 | 0.51 | 19 | 0.02 | 0.94 | 18 | 0.06 | 0.64 | 66 | -0.12 | 0.34 | 65 | -0.03 | 0.80 | 64 |
| M-CSF | **EV** | 0.23 | 0.38 | 18 | -0.03 | 0.92 | 19 | 0.22 | 0.41 | 18 | 0.04 | 0.75 | 56 | -0.08 | 0.57 | 55 | -0.03 | 0.81 | 55 |
|  | **Sol** | 0.34 | 0.20 | 18 | -0.07 | 0.79 | 19 | 0.20 | 0.46 | 18 | 0.11 | 0.39 | 67 | -0.06 | 0.65 | 66 | -0.02 | 0.86 | 65 |
| MIG | **EV** | -0.26 | 0.33 | 18 | -0.15 | 0.56 | 19 | -0.31 | 0.24 | 18 | -0.10 | 0.44 | 67 | -0.14 | 0.27 | 66 | -0.15 | 0.26 | 65 |
|  | **Sol** | 0.29 | 0.28 | 18 | 0.27 | 0.30 | 19 | 0.13 | 0.62 | 18 | -0.15 | 0.22 | 67 | -0.24 | 0.06 | 66 | -0.07 | 0.59 | 65 |
| MIP-1α | **EV** | 0.00 | 1.00 | 18 | 0.02 | 0.93 | 19 | 0.16 | 0.55 | 18 | 0.06 | 0.65 | 67 | 0.09 | 0.47 | 66 | 0.11 | 0.41 | 65 |
|  | **Sol** | -0.03 | 0.92 | 18 | 0.19 | 0.47 | 19 | 0.29 | 0.28 | 18 | -0.06 | 0.66 | 67 | -0.22 | 0.08 | 66 | -0.15 | 0.23 | 65 |
| MIP-1β | **EV** | -0.08 | 0.78 | 18 | 0.31 | 0.23 | 19 | 0.27 | 0.32 | 18 | -0.10 | 0.42 | 67 | 0.03 | 0.83 | 66 | 0.08 | 0.55 | 65 |
|  | **Sol** | -0.15 | 0.59 | 18 | 0.08 | 0.75 | 19 | 0.12 | 0.67 | 18 | -0.08 | 0.53 | 65 | -0.21 | 0.10 | 64 | -0.22 | 0.09 | 63 |
| MIP-3α | **EV** | -0.99 | 0.11 | 5 | -0.99 | 0.09 | 5 | -0.96 | 0.18 | 5 | 0.08 | 0.82 | 13 | -0.18 | 0.61 | 13 | -0.24 | 0.51 | 12 |
|  | **Sol** | -0.67 | 0.53 | 5 | 0.21 | 0.79 | 6 | 0.50 | 0.50 | 6 | 0.01 | 0.96 | 17 | -0.21 | 0.45 | 17 | 0.35 | 0.20 | 17 |
| MIP-3β | **EV** | -0.31 | 0.24 | 18 | -0.42 | 0.10 | 19 | -0.20 | 0.45 | 18 | -0.01 | 0.93 | 67 | 0.17 | 0.18 | 66 | 0.00 | 1.00 | 65 |
|  | **Sol** | 0.01 | 0.98 | 18 | -0.43 | 0.09 | 19 | -0.26 | 0.33 | 18 | 0.05 | 0.69 | 59 | -0.01 | 0.94 | 58 | -0.16 | 0.24 | 57 |
| PD-L1 | **EV** | -0.34 | 0.46 | 9 | -0.42 | 0.35 | 9 | -0.73 | 0.10 | 8 | 0.06 | 0.73 | 46 | -0.07 | 0.65 | 45 | -0.11 | 0.49 | 46 |
|  | **Sol** | 0.31 | 0.32 | 14 | -0.02 | 0.94 | 15 | 0.14 | 0.66 | 15 | -0.11 | 0.42 | 56 | -0.02 | 0.86 | 55 | -0.24 | 0.09 | 54 |
| TGF-β | **EV** | 0.22 | 0.47 | 15 | -0.22 | 0.46 | 16 | -0.02 | 0.95 | 15 | 0.19 | 0.20 | 51 | 0.06 | 0.67 | 51 | -0.05 | 0.73 | 50 |
|  | **Sol** | -0.23 | 0.62 | 9 | -0.24 | 0.56 | 10 | 0.12 | 0.79 | 9 | 0.11 | 0.54 | 36 | 0.02 | 0.90 | 36 | -0.01 | 0.97 | 34 |
| TNF-α | **EV** | -0.14 | 0.65 | 16 | -0.38 | 0.16 | 17 | -0.03 | 0.92 | 16 | 0.02 | 0.89 | 59 | -0.08 | 0.57 | 58 | -0.08 | 0.56 | 58 |
|  | **Sol** | 0.50 | 0.07 | 16 | -0.14 | 0.63 | 17 | -0.11 | 0.71 | 16 | 0.03 | 0.84 | 60 | -0.19 | 0.15 | 59 | -0.32 | 0.02 | 58 |
| TRAIL | **EV** | -0.25 | 0.36 | 18 | -0.37 | 0.14 | 19 | -0.23 | 0.40 | 18 | -0.02 | 0.89 | 67 | -0.08 | 0.52 | 66 | -0.11 | 0.41 | 65 |
|  | **Sol** | -0.13 | 0.63 | 18 | -0.55 | 0.02 | 19 | -0.49 | 0.05 | 18 | 0.10 | 0.45 | 67 | -0.08 | 0.56 | 66 | -0.11 | 0.38 | 65 |
| VEGF | **EV** | -0.10 | 0.72 | 17 | 0.10 | 0.72 | 18 | 0.20 | 0.48 | 17 | 0.02 | 0.90 | 53 | -0.01 | 0.96 | 52 | -0.09 | 0.54 | 51 |
|  | **Sol** | 0.09 | 0.79 | 14 | 0.03 | 0.93 | 15 | 0.29 | 0.35 | 14 | 0.20 | 0.15 | 54 | 0.02 | 0.90 | 53 | 0.01 | 0.95 | 52 |

During partial correlation analyses, cytokine data with concentrations below the LLOD and groups with n < 10 were removed from the analyses and Body Mass Index (BMI) and Karnofsky performance Status (KPS) score were controlled as covariates

**Abbreviations:** α = alpha, β = beta , CES-D = Center for Epidemiological Studies-Depression Scale, EV = extracellular vesicle, GSDS = General Sleep Disturbance Scale, IL = interleukin, GM-CSF = granulocyte-macrophage colony-stimulating factor, GRO-α = growth-regulated alpha, IFN-γ = interferon-γ, IP-10 = interferon-γ-induced protein, ITAC = interferon-inducible T-cell alpha chemoattractant, LLOD = lower limit of detection, M-CSF = macrophage colony-stimulating factor, MCP-1 = monocyte chemoattractant protein-1, MIG = monokine induced by IFN-γ, MIP = macrophage inflammatory protein, ND = not detected, PD-L1 = programmed death-ligand 1, RA = receptor antagonist, Sol = soluble, TGF-β = transforming growth factor beta, TNF-α = tumor necrosis factor-α, TRAIL = TNF- related apoptosis-inducing ligand, VEGF = vascular endothelial growth factor.

## Supplementary Table 4. Bivariate Correlation Analyses in Younger Cohort (age <60 years of age)

|  |  | **High Fatigue/Low Pain class** | | | | | | | | | **All Low class** | | | | | | | | |
| --- | --- | --- | --- | --- | --- | --- | --- | --- | --- | --- | --- | --- | --- | --- | --- | --- | --- | --- | --- |
| **Assay** | **Measure** | **CSD** | | | **GSDS** | | | **LFS** | | | **CSD** | | | **GSDS** | | | **LFS** | | |
| GM-CSF |  | **r** | **p** | **n** | **r** | **p** | **n** | **r** | **p** | **n** | **r** | **p** | **n** | **r** | **p** | **n** | **r** | **p** | **n** |
|  | **EV** | -0.14 | 0.38 | 41 | 0.12 | 0.47 | 42 | 0.13 | 0.41 | 42 | -0.01 | 0.96 | 59 | -0.14 | 0.29 | 59 | 0.00 | 0.98 | 58 |
| GRO-α | **Sol** | -0.27 | 0.06 | 49 | -0.01 | 0.93 | 50 | 0.00 | 0.99 | 50 | 0.23 | 0.07 | 67 | 0.07 | 0.57 | 66 | -0.08 | 0.55 | 67 |
|  | **EV** | -0.12 | 0.35 | 69 | -0.01 | 0.95 | 70 | 0.13 | 0.30 | 70 | 0.14 | 0.21 | 86 | -0.02 | 0.85 | 85 | 0.01 | 0.95 | 84 |
| IFN-γ | **Sol** | -0.13 | 0.32 | 61 | 0.02 | 0.90 | 62 | 0.09 | 0.47 | 62 | -0.05 | 0.67 | 87 | -0.06 | 0.59 | 86 | 0.05 | 0.66 | 85 |
|  | **EV** | -0.22 | 0.27 | 27 | 0.00 | 0.99 | 28 | 0.31 | 0.11 | 28 | 0.17 | 0.30 | 40 | 0.03 | 0.86 | 40 | 0.15 | 0.35 | 40 |
| IL-10 | **Sol** | -0.20 | 0.32 | 29 | 0.20 | 0.31 | 30 | 0.07 | 0.71 | 30 | 0.09 | 0.58 | 40 | -0.33 | 0.04 | 40 | -0.24 | 0.14 | 40 |
|  | **EV** | -0.87 | 0.05 | 6 | -0.35 | 0.57 | 6 | -0.04 | 0.95 | 6 | -0.26 | 0.74 | 5 | 0.95 | 0.05 | 5 | -0.29 | 0.64 | 6 |
| IL-12p70 | **Sol** | -0.16 | 0.74 | 8 | -0.38 | 0.35 | 9 | 0.11 | 0.80 | 9 | 0.53 | 0.04 | 17 | -0.10 | 0.71 | 17 | -0.03 | 0.91 | 17 |
|  | **EV** | -0.05 | 0.70 | 64 | 0.03 | 0.79 | 65 | 0.15 | 0.24 | 65 | -0.05 | 0.66 | 88 | -0.06 | 0.57 | 87 | 0.09 | 0.39 | 86 |
| IL-15 | **Sol** | -0.07 | 0.66 | 45 | 0.08 | 0.63 | 45 | 0.23 | 0.14 | 46 | 0.00 | 0.97 | 58 | 0.15 | 0.27 | 57 | 0.07 | 0.59 | 57 |
|  | **EV** | -0.04 | 0.82 | 39 | -0.26 | 0.11 | 39 | 0.21 | 0.20 | 40 | 0.10 | 0.49 | 56 | -0.02 | 0.88 | 55 | -0.05 | 0.69 | 57 |
| IL-16 | **Sol** | 0.01 | 0.96 | 76 | 0.08 | 0.47 | 77 | -0.10 | 0.38 | 77 | 0.04 | 0.71 | 103 | -0.11 | 0.29 | 102 | -0.03 | 0.78 | 101 |
|  | **EV** | -0.05 | 0.67 | 81 | -0.17 | 0.13 | 82 | 0.12 | 0.28 | 82 | -0.01 | 0.92 | 109 | 0.09 | 0.37 | 108 | 0.17 | 0.08 | 107 |
| IL-17 | **Sol** | -0.02 | 0.89 | 81 | -0.11 | 0.32 | 82 | 0.18 | 0.10 | 82 | 0.09 | 0.35 | 108 | 0.13 | 0.17 | 107 | 0.05 | 0.58 | 106 |
|  | **EV** | 0.22 | 0.33 | 23 | -0.14 | 0.53 | 24 | 0.24 | 0.27 | 24 | 0.03 | 0.85 | 38 | -0.09 | 0.58 | 38 | -0.11 | 0.52 | 37 |
| IL-18 | **Sol** | -0.31 | 0.13 | 26 | 0.30 | 0.14 | 27 | -0.01 | 0.95 | 27 | -0.02 | 0.89 | 41 | 0.04 | 0.82 | 41 | 0.23 | 0.15 | 41 |
|  | **EV** | -0.13 | 0.34 | 62 | -0.27 | 0.03 | 63 | 0.04 | 0.77 | 63 | -0.05 | 0.61 | 90 | 0.08 | 0.44 | 89 | -0.05 | 0.62 | 88 |
| IL-1RA | **Sol** | -0.07 | 0.52 | 81 | -0.16 | 0.16 | 82 | 0.08 | 0.46 | 82 | 0.03 | 0.77 | 109 | 0.06 | 0.53 | 108 | 0.11 | 0.26 | 107 |
|  | **EV** | -0.09 | 0.48 | 60 | -0.17 | 0.21 | 60 | 0.17 | 0.21 | 60 | -0.10 | 0.35 | 84 | -0.10 | 0.35 | 83 | 0.01 | 0.92 | 82 |
| IL-1α | **Sol** | 0.01 | 0.96 | 59 | -0.01 | 0.96 | 60 | 0.17 | 0.19 | 60 | -0.07 | 0.52 | 86 | 0.00 | 0.99 | 85 | -0.08 | 0.48 | 84 |
|  | **EV** | -0.23 | 0.07 | 64 | -0.27 | 0.03 | 64 | 0.15 | 0.25 | 64 | -0.10 | 0.38 | 81 | -0.18 | 0.11 | 80 | -0.09 | 0.41 | 79 |
| IL-1β | **Sol** | -0.09 | 0.48 | 64 | 0.11 | 0.40 | 65 | 0.18 | 0.17 | 64 | 0.00 | 1.00 | 88 | 0.09 | 0.39 | 87 | 0.03 | 0.82 | 86 |
|  | **EV** | -0.11 | 0.43 | 59 | -0.08 | 0.54 | 59 | 0.14 | 0.31 | 58 | 0.21 | 0.06 | 83 | 0.02 | 0.86 | 82 | 0.14 | 0.22 | 81 |
| IL-2 | **Sol** | -0.14 | 0.28 | 61 | -0.11 | 0.41 | 62 | 0.08 | 0.54 | 62 | 0.21 | 0.06 | 81 | 0.06 | 0.58 | 80 | -0.06 | 0.62 | 81 |
|  | **EV** | -0.07 | 0.65 | 46 | -0.17 | 0.26 | 46 | 0.15 | 0.31 | 47 | 0.20 | 0.09 | 75 | 0.10 | 0.41 | 74 | 0.04 | 0.74 | 74 |
| IL-22 | **Sol** | -0.14 | 0.33 | 49 | 0.05 | 0.71 | 50 | 0.01 | 0.96 | 50 | 0.07 | 0.56 | 65 | 0.10 | 0.43 | 64 | 0.11 | 0.38 | 65 |
|  | **EV** | -0.46 | 0.01 | 33 | -0.30 | 0.09 | 34 | 0.08 | 0.67 | 34 | -0.13 | 0.37 | 48 | -0.04 | 0.79 | 48 | 0.19 | 0.20 | 48 |
| IL-4 | **Sol** | -0.19 | 0.23 | 42 | 0.11 | 0.47 | 43 | -0.17 | 0.28 | 43 | 0.24 | 0.05 | 65 | 0.01 | 0.93 | 65 | -0.06 | 0.64 | 63 |
|  | **EV** | -0.14 | 0.30 | 60 | -0.14 | 0.28 | 61 | 0.11 | 0.41 | 61 | -0.11 | 0.33 | 84 | 0.06 | 0.57 | 83 | 0.02 | 0.88 | 83 |
| IL-6 | **Sol** | -0.02 | 0.92 | 46 | 0.17 | 0.25 | 47 | 0.19 | 0.19 | 47 | 0.03 | 0.84 | 60 | -0.07 | 0.62 | 60 | 0.00 | 1.00 | 58 |
|  | **EV** | -0.18 | 0.21 | 51 | -0.14 | 0.34 | 51 | 0.09 | 0.53 | 52 | -0.02 | 0.90 | 66 | -0.03 | 0.84 | 65 | -0.13 | 0.31 | 64 |
| IL-7 | **Sol** | -0.12 | 0.46 | 42 | 0.19 | 0.22 | 43 | 0.06 | 0.69 | 43 | 0.02 | 0.89 | 56 | 0.07 | 0.62 | 56 | 0.16 | 0.26 | 54 |
|  | **EV** | -0.20 | 0.14 | 54 | -0.20 | 0.14 | 54 | 0.07 | 0.64 | 55 | -0.16 | 0.15 | 80 | -0.01 | 0.95 | 79 | -0.01 | 0.95 | 79 |
| IL-8 | **Sol** | -0.12 | 0.33 | 74 | 0.14 | 0.24 | 74 | 0.18 | 0.12 | 75 | 0.06 | 0.56 | 104 | 0.03 | 0.78 | 103 | 0.05 | 0.63 | 102 |
|  | **EV** | -0.03 | 0.81 | 53 | -0.12 | 0.40 | 54 | 0.19 | 0.18 | 54 | -0.08 | 0.51 | 80 | -0.06 | 0.58 | 80 | -0.02 | 0.89 | 78 |
| IP-10 | **Sol** | -0.07 | 0.58 | 67 | -0.02 | 0.89 | 68 | 0.05 | 0.66 | 68 | -0.08 | 0.42 | 105 | 0.02 | 0.85 | 104 | 0.04 | 0.66 | 103 |
|  | **EV** | -0.01 | 0.93 | 77 | 0.11 | 0.36 | 77 | 0.35 | 0.00 | 77 | -0.11 | 0.25 | 105 | 0.03 | 0.79 | 104 | 0.08 | 0.42 | 103 |
| ITAC | **Sol** | 0.05 | 0.69 | 81 | 0.14 | 0.22 | 82 | 0.28 | 0.01 | 82 | 0.01 | 0.95 | 109 | 0.11 | 0.25 | 108 | 0.09 | 0.37 | 107 |
|  | **EV** | -0.20 | 0.08 | 81 | -0.17 | 0.14 | 82 | 0.13 | 0.25 | 82 | -0.03 | 0.79 | 109 | 0.03 | 0.76 | 108 | 0.07 | 0.51 | 107 |
| MCP-1 | **Sol** | -0.09 | 0.43 | 81 | -0.03 | 0.76 | 82 | 0.07 | 0.52 | 82 | 0.05 | 0.59 | 109 | -0.01 | 0.89 | 108 | -0.02 | 0.82 | 107 |
|  | **EV** | -0.14 | 0.64 | 14 | -0.10 | 0.73 | 15 | -0.10 | 0.73 | 15 | 0.00 | 0.99 | 20 | -0.19 | 0.44 | 20 | -0.25 | 0.28 | 21 |
| M-CSF | **Sol** | -0.15 | 0.17 | 81 | -0.01 | 0.93 | 82 | 0.15 | 0.18 | 82 | -0.10 | 0.30 | 109 | 0.08 | 0.39 | 108 | 0.05 | 0.58 | 107 |
|  | **EV** | -0.24 | 0.04 | 74 | -0.24 | 0.04 | 75 | 0.12 | 0.31 | 75 | -0.07 | 0.49 | 101 | -0.02 | 0.86 | 100 | 0.01 | 0.96 | 100 |
| MIG | **Sol** | -0.07 | 0.56 | 81 | 0.03 | 0.76 | 82 | 0.17 | 0.12 | 82 | -0.03 | 0.74 | 109 | 0.06 | 0.53 | 108 | -0.05 | 0.59 | 107 |
|  | **EV** | -0.36 | 0.00 | 81 | -0.11 | 0.34 | 82 | -0.06 | 0.57 | 82 | -0.09 | 0.37 | 109 | -0.07 | 0.45 | 108 | -0.09 | 0.36 | 107 |
| MIP-1α | **Sol** | -0.05 | 0.68 | 81 | 0.08 | 0.48 | 82 | 0.20 | 0.07 | 82 | -0.01 | 0.95 | 109 | -0.05 | 0.59 | 108 | 0.05 | 0.65 | 107 |
|  | **EV** | 0.05 | 0.66 | 81 | 0.06 | 0.59 | 82 | 0.12 | 0.27 | 82 | 0.00 | 0.96 | 109 | 0.03 | 0.78 | 108 | -0.02 | 0.85 | 107 |
| MIP-1β | **Sol** | 0.02 | 0.88 | 80 | 0.01 | 0.94 | 81 | 0.14 | 0.21 | 81 | 0.05 | 0.58 | 109 | 0.05 | 0.61 | 108 | 0.08 | 0.39 | 107 |
|  | **EV** | 0.00 | 1.00 | 81 | -0.05 | 0.69 | 82 | 0.16 | 0.15 | 82 | -0.01 | 0.91 | 109 | 0.07 | 0.50 | 108 | 0.10 | 0.29 | 107 |
| MIP-3α | **Sol** | 0.02 | 0.88 | 81 | 0.08 | 0.46 | 82 | 0.11 | 0.34 | 82 | 0.05 | 0.61 | 109 | 0.06 | 0.56 | 108 | 0.11 | 0.25 | 107 |
|  | **EV** | -0.22 | 0.45 | 15 | -0.15 | 0.60 | 16 | -0.21 | 0.46 | 16 | 0.28 | 0.29 | 17 | 0.25 | 0.36 | 17 | -0.36 | 0.19 | 16 |
| MIP-3β | **Sol** | -0.49 | 0.02 | 24 | -0.22 | 0.30 | 25 | -0.11 | 0.59 | 25 | -0.02 | 0.91 | 31 | -0.36 | 0.05 | 31 | -0.23 | 0.23 | 31 |
|  | **EV** | -0.01 | 0.95 | 81 | 0.09 | 0.44 | 82 | 0.24 | 0.03 | 82 | 0.00 | 0.98 | 109 | 0.12 | 0.22 | 108 | 0.01 | 0.93 | 107 |
| PD-L1 | **Sol** | -0.04 | 0.74 | 69 | -0.08 | 0.52 | 69 | 0.15 | 0.22 | 69 | -0.11 | 0.28 | 94 | 0.13 | 0.22 | 93 | 0.12 | 0.26 | 92 |
|  | **EV** | -0.07 | 0.61 | 52 | -0.17 | 0.24 | 52 | 0.01 | 0.97 | 52 | 0.02 | 0.85 | 65 | -0.07 | 0.61 | 64 | -0.03 | 0.81 | 64 |
| TGF-β | **Sol** | -0.13 | 0.30 | 67 | -0.21 | 0.08 | 68 | 0.13 | 0.30 | 69 | -0.08 | 0.45 | 89 | 0.08 | 0.46 | 88 | -0.16 | 0.13 | 87 |
|  | **EV** | -0.08 | 0.56 | 54 | 0.18 | 0.20 | 55 | 0.23 | 0.09 | 55 | 0.03 | 0.76 | 84 | -0.05 | 0.62 | 84 | 0.00 | 0.97 | 82 |
| TNF-α | **Sol** | -0.36 | 0.03 | 39 | 0.04 | 0.80 | 40 | 0.04 | 0.82 | 40 | 0.12 | 0.36 | 57 | 0.05 | 0.73 | 57 | 0.00 | 0.98 | 57 |
|  | **EV** | -0.16 | 0.18 | 73 | -0.01 | 0.90 | 74 | 0.18 | 0.12 | 75 | -0.02 | 0.87 | 92 | -0.15 | 0.17 | 91 | 0.10 | 0.37 | 90 |
| TRAIL | **Sol** | -0.12 | 0.36 | 64 | 0.19 | 0.13 | 64 | 0.10 | 0.42 | 64 | -0.02 | 0.84 | 90 | 0.02 | 0.84 | 89 | -0.02 | 0.83 | 88 |
|  | **EV** | 0.01 | 0.96 | 81 | 0.26 | 0.02 | 82 | 0.20 | 0.07 | 82 | -0.06 | 0.53 | 108 | -0.05 | 0.62 | 107 | 0.01 | 0.92 | 106 |
| VEGF | **Sol** | 0.10 | 0.39 | 81 | 0.20 | 0.07 | 82 | 0.20 | 0.07 | 82 | 0.02 | 0.81 | 109 | 0.04 | 0.70 | 108 | -0.05 | 0.62 | 107 |
|  | **EV** | -0.10 | 0.44 | 62 | -0.05 | 0.72 | 63 | 0.21 | 0.10 | 63 | -0.09 | 0.40 | 88 | -0.02 | 0.85 | 87 | 0.12 | 0.29 | 86 |
|  | **Sol** | -0.12 | 0.34 | 65 | -0.20 | 0.12 | 65 | 0.13 | 0.29 | 65 | 0.08 | 0.46 | 84 | 0.12 | 0.30 | 83 | 0.18 | 0.10 | 82 |

During partial correlation analyses, cytokine data with concentrations below the LLOD and groups with n < 10 were removed from the analyses and Karnofsky performance Status (KPS) score was controlled as a covariate.

**Abbreviations:** α = alpha, β = beta , CES-D = Center for Epidemiological Studies-Depression Scale, EV = extracellular vesicle, GSDS = General Sleep Disturbance Scale, IL = interleukin, GM-CSF = granulocyte-macrophage colony-stimulating factor, GRO-α = growth-regulated alpha, IFN-γ = interferon-γ, IP-10 = interferon-γ-induced protein, ITAC = interferon-inducible T-cell alpha chemoattractant, LLOD = lower limit of detection, M-CSF = macrophage colony-stimulating factor, MCP-1 = monocyte chemoattractant protein-1, MIG = monokine induced by IFN-γ, MIP = macrophage inflammatory protein, PD-L1 = programmed death-ligand 1, RA = receptor antagonist, Sol = soluble, TGF-β = transforming growth factor beta, TNF-α = tumor necrosis factor-α, TRAIL = TNF- related apoptosis-inducing ligand, VEGF = vascular endothelial growth factor.

## References

Mercurio, V., Fitzgerald, W., Vanpouille, C., Molodtsov, I., and Margolis, L. (2021). Mechanisms of residual immune activation in HIV-1-infected human lymphoid tissue ex vivo. *AIDS (London, England)* 35(8)**,** 1179-1190.

Senn, S., Holford, N., and Hockey, H. (2012). The ghosts of departed quantities: approaches to dealing with observations below the limit of quantitation. *Statistics in medicine* 31(30)**,** 4280-4295.
